# A plasmid toolset for CRISPR‐mediated genome editing and CRISPRi gene regulation in *Escherichia coli*

**DOI:** 10.1111/1751-7915.13780

**Published:** 2021-03-12

**Authors:** Adrian J. Jervis, Erik K.R. Hanko, Mark S. Dunstan, Christopher J. Robinson, Eriko Takano, Nigel S. Scrutton

**Affiliations:** ^1^ Manchester Centre for Fine and Speciality Chemicals (SYNBIOCHEM) Manchester Institute of Biotechnology University of Manchester Manchester M1 7DN UK

## Abstract

CRISPR technologies have become standard laboratory tools for genetic manipulations across all kingdoms of life. Despite their origins in bacteria, the development of CRISPR tools for engineering bacteria has been slower than for eukaryotes; nevertheless, their function and application for genome engineering and gene regulation via CRISPR interference (CRISPRi) has been demonstrated in various bacteria, and adoption has become more widespread. Here, we provide simple plasmid‐based systems for genome editing (gene knockouts/knock‐ins, and genome integration of large DNA fragments) and CRISPRi in *E. coli* using a CRISPR‐Cas12a system. The described genome engineering protocols allow markerless deletion or genome integration in just seven working days with high efficiency (> 80% and 50%, respectively), and the CRISPRi protocols allow robust transcriptional repression of target genes (> 90%) with a single cloning step. The presented minimized plasmids and their associated design and experimental protocols provide efficient and effective CRISPR‐Cas12 genome editing, genome integration and CRISPRi implementation. These simple‐to‐use systems and protocols will allow the easy adoption of CRISPR technology by any laboratory.

## Graphical abstract

Minimized plasmids and their associated design and experimental protocols have been developed to provide efficient and effective CRISPR‐Cas12 genome editing, genome integration and CRISPRi implementation. These simple‐to‐use systems and protocols will allow the easy adoption of CRISPR technology by any laboratory.

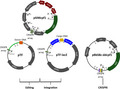

## Introduction

Genetic engineering techniques derived from clustered regularly interspaced short palindromic repeats (CRISPR) and Cas nucleases have revolutionized genome editing in a large range of organisms. The most common and successful CRISPR applications have been for genome engineering and gene regulation. CRISPR systems for genome engineering consist of two key components: a Cas nuclease (e.g. Cas9 and Cas12a/Cpf1); and a CRISPR array of alternating repeat and spacer DNA sequences (reviewed by Jiang and Marraffini, 2015b). The CRISPR array is transcribed as a long precursor, which is then processed by cleavage in the repeat regions to produce individual target‐specific CRISPR RNAs (crRNA), which direct the Cas nuclease to complementary double‐stranded DNA (dsDNA) targets, where the Cas nuclease creates a double stranded break (DSB). Natural or directed recombinant repair of this DSB can result in edits in the target dsDNA.

For the type II CRISPR systems (e.g. CRISPR‐Cas9), the crRNA is combined with a trans‐activating RNA (tracrRNA) to produce a guide RNA (gRNA). This system has been manipulated to enable the design of a single small guide RNA (sgRNA), which contains both the gRNA and the tracrRNA as a single transcript, without the need for crRNA processing by accessory Cas enzymes (Jinek *et al*., 2012). In the case of CRISPR‐Cas12a (type V) systems, the Cas12a is a dual‐function nuclease that processes the crRNA and cleaves dsDNA targets, requiring no accessory Cas proteins (Fonfara et al., 2016). By creating *de novo* engineered small guide RNAs (sgRNA) or CRISPR arrays, users can target genome cleavage to any sequence that contains the prerequisite 3–4 bp protospacer adjacent motif (PAM) by modifying the spacer region(s). Supply of user‐defined donor DNA (dDNA), sharing homology with the cleavage site, in recombination‐competent cells, allows repair of the dsDNA break, replacing the target sequence with the dDNA in the process (Jiang *et al*., 2015a). Without repair, the cell will die, allowing for a ‘live–dead’ selection method (Jiang *et al*., 2015a; Cui and Bikard, 2016). CRISPR genome editing has major advantages over conventional methods including rapid protocols, scarless editing, no requirement for selection markers and the possibility of multiplexed editing (Jiang *et al*., 2015a; Ao *et al*., 2018).

CRISPRi is a further development of CRISPR technology for gene regulation, in which ‘dead’ nuclease mutants (e.g. dCas9 or ddCpf1) are employed (Qi *et al*., 2013; Fontana *et al*., 2018). The dead nucleases maintain their DNA binding capability and can be targeted to open reading frames (ORFs) or promoter regions, where they allosterically block transcription, resulting in transcriptional silencing of genes (Qi *et al*., 2013). This offers advantages over basic CRISPR‐based knockout technology, including the ability to target essential genes (Wiktor *et al*., 2016; Rousset *et al*., 2018), tunability (Fontana *et al*., 2018) and multiplexing (Zetsche *et al*., 2017; Zhang *et al*., 2017) on plasmid‐based systems. These properties make CRISPRi a very powerful, yet simple, rapid and cost‐effective gene regulation technology.

The most dramatic benefits of CRISPR technologies have been realized in eukaryotic organisms, particularly mammalian cells. In contrast, the uptake for bacterial systems, including *Escherichia coli*, has been limited, possibly because many effective engineering systems already exist for bacteria employing phages and transposons (Hayes, 2003; Akhverdyan *et al*., 2011), or recombineering (Yoon *et al*., 1998), and technical hurdles discourage the adoption of new techniques.

Since the demonstration of CRISPR‐Cas function, several systems have been described for use in *E. coli* genome editing (Li *et al*., 2015; Pyne *et al*., 2015; Jiang *et al*., 2015a; Ao *et al*., 2018) and CRISPRi (Wu *et al*., 2015; Kim *et al*., 2016; Li *et al*., 2016; Wiktor *et al*., 2016; Yan *et al*., 2017; Zhang *et al*., 2017; Dwidar and Yokobayashi, 2019; Roghanian *et al*., 2019). To date, many of the key publications in *E. coli* CRISPR research describe the mechanisms by which the technology functions or the development of tools for creating gene knockouts or small integrations or CRISPRi. We and others have not found it simple to adopt some of these methods and so here we describe new CRISPR‐Cas12a plasmids, for both genome editing, including integration of large DNA fragments to the genome, and CRISPRi in *E. coli* along with detailed protocols for their easy modification and implementation. We envisage broad application and clear design and laboratory protocols will facilitate the adoption of this transformative technology in any laboratory.

## Results

### Construction of a CRISPR‐Cas12a editing system

**Fig. 1:**
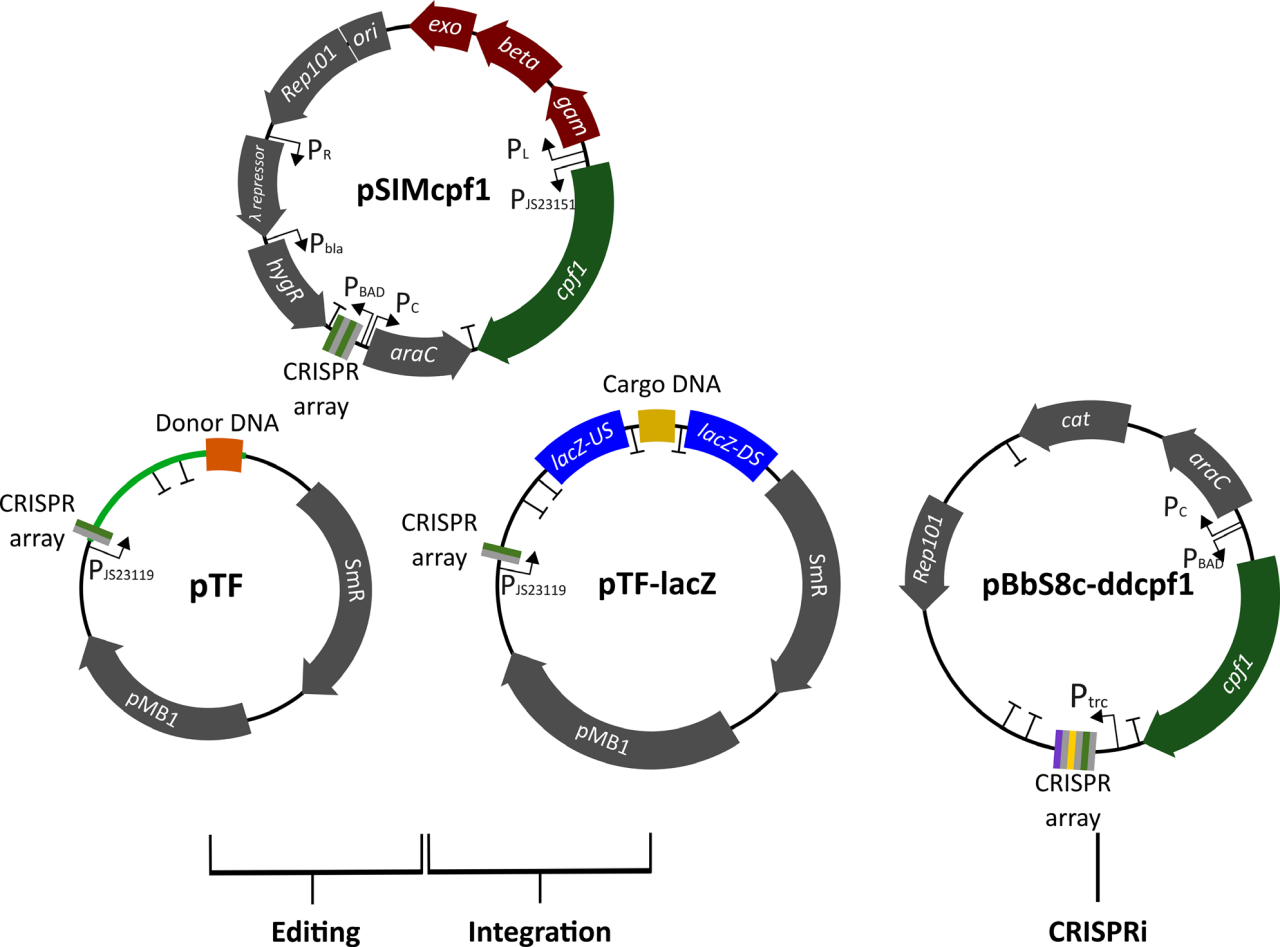
Plasmid toolset for CRISPR genome editing, genome integration and CRISPR interference. For CRISPR editing (gene knockout/knock‐in or base editing), a combination of pTF and pSIM*cpf1* are used. The green section of pTF is designed as a target‐specific cassette, which encodes both a bespoke CRISPR array and dDNA, and this can be commercially synthesized. For genome integration of large cargo DNA, pTF‐*lacZ* is used in combination with pSIMcpf1. With this plasmid, the cargo DNA will be integrated at the *lacZ* gene locus on the chromosome. For CRISPRi repression of target genes, pBbS8c‐*ddcpf1* is modified to target a gene(s) of interest by inserting a bespoke CRISPR array.

We chose to build a CRISPR‐Cas12a system (over CRISPR‐Cas9) because of the relative ease of constructing CRISPR arrays. For CRISPR‐Cas12a systems, a single transcript of one repeat and one spacer sequence (42 bp) is required as a CRISPR array and this can be extended to target additional sequences by adding extra repeat/spacer sequences in increments (each 42 bp) on the same transcript. For CRISPR‐Cas9, only 20 bp of the 102 bp sgRNA needs to be modified but a new transcriptional unit needs to be added for each target. The plasmid design was based on the editing system described by Jiang et al. (2015a) for use in *E. coli*, employing CRISPR‐Cas9 in combination with Lambda Red recombineering. Accordingly, two plasmids were designed and constructed (Fig. 1). The first, pSIM*cpf1*, is modified from the Lambda Red recombineering plasmid, pSIM18 (Chan *et al*., 2007). It encodes the Lambda Red recombination genes under the control of the temperature‐responsive promoter P_L_, a Cas12a gene (*cpf1* from *Acidaminococcus sp*. BV3L6) under control of a constitutive promoter (P_JS23150_), and a P_BAD_‐regulated CRISPR array which targets the pMB1 origin of replication of the second plasmid pTF (which contains all the target‐specific elements). To construct pTF, pTargetF (Jiang *et al*., 2015a) was modified to replace the Cas9 sgRNA with a Cas12a CRISPR array under constitutive expression, and to include an optional dDNA cloning site for genome integrations. After successful editing, pTF can be removed from cells by inducing the pSIM*cpf1* CRISPR array to cleave the pTF replication origin, and then pSIM*cpf1* itself can be cured from the cell by growth at elevated temperatures (Jiang *et al*., 2015a).

To modify pTF for a new target, a bespoke CRISPR array and dDNA must be introduced. To simplify this process, a single DNA cassette was designed to include both the CRISPR array (Table S2) and dDNA (Fig. S1), and overlapping sequences to be cloned directly into PCR‐linearized pTF using recombination cloning with the InFusion system (Takara Bio Inc., Kusatsu, Japan). To tailor the cassette to the user’s requirements, only the CRISPR array spacer and dDNA sequences need to be altered. The size of the cassette will vary depending on the size of the dDNA but for a single gene knockout using two 50 bp homologous arms, as per standard recombineering protocols (Datesenko and Wanner, 2000), the entire cassette will be only 368 bp in size, for which commercial DNA synthesis is cost effective (< $65) for most laboratory budgets. For the initial test of this system, a cassette was cloned into pTF to target and inactivate the non‐essential *ahpC* gene through the in‐frame insertion of an *rfp* reporter gene. For this first test, and to ensure high recombination efficiency (Muyrers *et al*., 2000), we used extended 200 bp homologous arms for recombination which resulted in a total cassette size of 1.3 kb.

The Lambda Red genes were induced in *E. coli* MG1655 carrying pSIM*cpf1* by elevating the temperature of the growth media, then these cells were transformed with either pTF (no spacer, no dDNA), pTF‐*ahpC* (*ahpC* guide, no dDNA) or pTF‐*ahpC*‐*rfp* (*ahpC* spacer and *rfp* dDNA), and plated on agar selective for both plasmids (Fig. S2). When both the guide and *cpf1* were present there was a 3.8 x 10^3^ drop in colony forming units (CFUs) compared to samples with no guide, indicating successful cleavage of the genomic DNA (Table 1) and agreeing with previously published decreases in transformation efficiency (Jiang *et al*., 2015a). A similar number of CFUs were observed both with and without dDNA, but RFP fluorescence was observed only for those colonies transformed with pTF‐*ahpC*‐*rfp*. PCR using primers designed to flank the recombination site confirmed that 31/36 (86%) of these colonies had *rfp* correctly integrated at the *ahpC* locus (Table 1). A positive clone was selected at random and cured of both plasmids as follows: incubation at 30°C for 6 h in LB media supplemented with hygromycin (to maintain pSIM*cpf1*) and L‐arabinose (to induce CRISPR‐mediated knockout of pTF plasmid); then subculture into LB without antibiotics and incubated at 37°C for 16 h to remove pSIM*cpf1*. Each step was successful in curing the respective plasmids in over 99% of colonies, and plasmid‐free cells were recovered in under 24 h without the need for iterative testing of plasmid loss.

**Table 1:** Efficiency of genome editing in *E. coli*.

Strain	Target mutation	Activity	Relative CFU count^a^	Correct clone
MG1655	*ahpC::rfp*; 1.3 kb	Integration	3.0 × 10^‐4^	31/36 (86%)
MG1655	Δ*tyrR*; 1.5 kb	Deletion	1.77 × 10^‐4^	4/4 (100%)
DH5α	Δ*tyrR*; 1.5 kb	Deletion	6.0 × 10^‐5^	3/4 (75%)
MG1655	*lacZ*::P_JS23119_‐*rfp*; 2kb	Integration	7.1 × 10^‐2^	7/8 (86%)
MG1655	*lacZ*::5753; 8.4 kb	Integration	1.1 × 10^‐4^	4/8 (50%)
DH5α	*lacZ*::5753 8.4 kb	Integration	6.5 × 10^‐4^	3/8 (38%)

Different editing functions are displayed for either genomic integrations (with associated total DNA integrated) or gene deletions at different targets and using different *E. coli* host.

^a^ The fraction of colony‐forming units (CFUs) observed compared to a control with no spacer sequence, and the number of clones screened with the correct edit are both shown.

To test the methodology for producing markerless gene deletions, a pTF cassette was designed with a CRISPR sequence to target the *tyrR* gene and a dDNA encoding 50 bp homologous arms to delete all but the start codon and the final seven codons at the 3′ gene terminus of *tyrR*. These homologous arms were designed following the same strategy described previously for Lambda Red recombineering (Baba *et al*., 2006; Robinson *et al*., 2020). The pTF‐*tyrR* plasmid was used to edit both *E. coli* MG1655 and *E. coli* DH5α, and the results showed a similar fold (~ 10^3^) reduction in CFUs and high editing efficiency to that observed for the *ahpC*::*rfp* integration above (Table 1).

### Integration of large DNA fragments into the genome

A common aim for microbial genome editing is to integrate large DNA fragments, such as whole metabolic pathways, into the genome in a single step; however, integration of DNA fragments becomes more challenging as their size increases (Kuhlman and Cox, 2010). To this end, a variant of pTF was designed (pTF‐*lacZ*) for the efficient delivery of cargo DNA to the *lacZ* locus of the *E. coli* genome. pTF‐*lacZ* was designed to include 500 bp upstream and downstream recombination arms to increase the efficiency of recombination (Muyrers *et al*., 2000; Yu *et al*., 2000). The two *lacZ* homologous arms are separated by transcriptional terminators designed to flank and transcriptionally insulate cargo DNA sequences (Fig. 1). To add cargo DNA to pTF‐*lacZ* it is linearized by inverse PCR (Table S1), whilst the cargo sequence is amplified with PCR primers to include homology to the linearized pTF‐*lacZ*, facilitating rapid and standardized cloning.

Initial tests of pTF‐*lacZ* efficacy used a 2 kb *rfp* reporter gene under the control of constitutive promoter P_JS23100_ as the cargo. Editing efficiencies (86%) were comparable to those of single gene deletions or insertions at the *ahpC*/*tyrR* loci (Table 1). To investigate the editing efficiency for larger cargo DNA sequences, we used a previously constructed plasmid‐borne construct (SBC005753) designed to boost levels of tyrosine in *E. coli* (Robinson *et al*., 2020). The inducible construct employs *lac*‐based promoters to drive three refactored *E. coli* genes (*ppsA*, aroG*, tyrA*) and, in total with its *lacI* repressor gene, the total DNA cargo size is 7.9 kb. This was cloned into pTF‐*lacZ* to create plasmid pTF‐*lacZ*‐5753, which was used with pSIM*cpf1* to edit the genomes of *E. coli* MG1655 and DH5α strains using the same workflow described above for single gene knockouts. A reduction in CFUs was observed but in comparison to the integration of the small *rfp* expression cassette, the proportion of correct clones identified was lower (*lacZ*::5753 and *lacZ*::P_JS23119_‐*rfp* were 38–50% and 86%, respectively; Table 1). Colony PCR screening was more challenging due to the large size of the integration and so screening had to be performed using purified genomic DNA as the template, reducing the number of colonies screened and extending the protocol by 1 day. Both the upstream and downstream integration junctions and the complete integration were checked for each colony by PCR, using primers designed for genome sequences flanking the inserted DNA (Fig. S3). The *E. coli* integrant strains were also checked for increased tyrosine production under induction conditions (Robinson *et al*., 2020). The total size of integrated DNA (including the homologous recombination arms) was 8.4 kb, surpassing most previous reports for one‐step delivery to the *E. coli* chromosome (Kuhlman and Cox, 2010; Sabri *et al*., 2013; St‐Pierre *et al*., 2013).

### Construction and testing of a Cas12a CRISPRi system

**Fig. 2:**
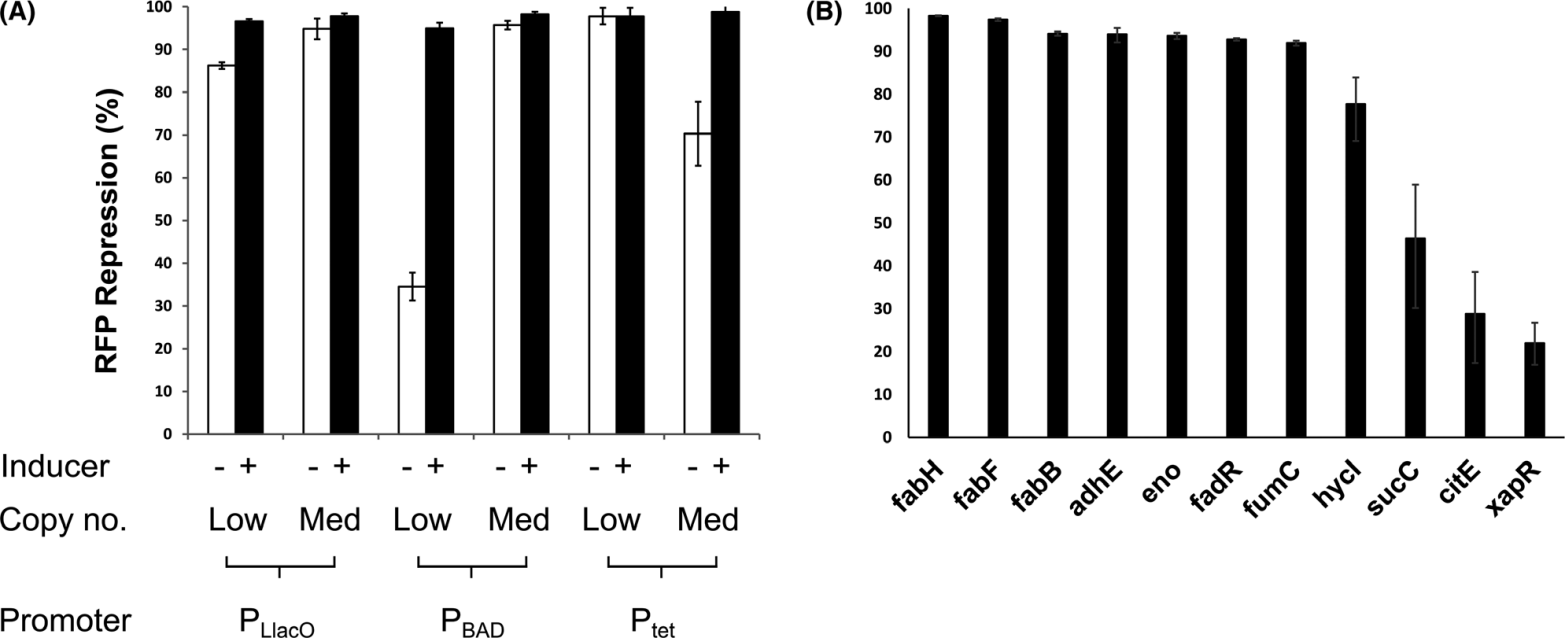
Repression of *rfp* expression by CRISPRi plasmid variants. A. Six different CRISPRi systems targeting *rfp* were co‐expressed with a constitutive *rfp* expression plasmid (25‐30 copies) in *E. coli* DH5α. The CRISPRi plasmids, with different origins of replication for low or medium copy numbers, were tested with and without induction from three different promoters. B. Gene‐specific spacer regions were designed to individually target 11 different chromosomal genes, with guide efficiency assessed by RT‐qPCR. Samples were prepared from cells in the late logarithmic growth phase and results are reported as percentage repression relative to controls which contain no spacer sequence. Each sample was grown in biological triplicates and all qPCR reactions were performed in technical triplicates.

For Cpf1, a single point mutation (E993A) has been described which creates a DNA nuclease deficient mutant (ddCpf1; RNA nuclease competent), which has been used for CRISPRi applications (Zetche et al., 2017; Zhang *et al*., 2017). We introduced this mutation into our *cpf1* gene and added a downstream CRISPR array, regulated by a P_trc_ promoter, with either no spacer sequence, or a spacer sequence targeting the 5’ end of an *rfp* reporter gene. This construct was introduced into vector pBbB2c‐*rfp* (to replace the *rfp* gene) creating plasmid pBbB2c‐*ddcpf1‐rfp* which is subject to differential regulation by IPTG (CRISPR array) and anhydrotetracycline (aTet) (*ddcpf1*). To test functionality, the CRISPRi plasmids were co‐transformed into *E. coli* DH5α with constitutive expression plasmid pBbE11a‐*rfp* (Jervis *et al*., 2019), and grown with varying concentrations of the two inducers. Endpoint readings of cell density and fluorescence were taken and showed the system was capable of tightly repressing expression of RFP in the presence of the *rfp* spacer and aTet (Fig. S4). There was some repression in the absence of inducer but more significantly, we observed a small amount of repression when there were inducers even with no spacer sequence. The cell density was reduced with increasing concentration of aTet (*ddcpf1* expression), particularly in cultures with an *rfp* spacer. Toxicity of Cas nucleases is a well‐known phenomenon (Jiang *et al*., 2017; Zhang *et al*., 2017; Cho *et al*., 2018) and is likely responsible for the reduction in *rfp* expression in the absence of inducer and spacer. To reduce the toxicity, but maintain activity we sub‐cloned the system into a range of expression vectors (Lee *et al*., 2011) with different inducible promoters and copy numbers (Table S5); one example (pBbS8c‐*ddcpf1*) is shown in Figure 1. All of the constructs resulted in over 90% repression of RFP fluorescence when *ddcpf1* and the *rfp* spacer were induced, compared to a control plasmid in which there was no spacer present (Fig. 2A). Significant reductions in fluorescence were observed even in the absence of inducer but was particularly pronounced in plasmids with either P_BAD_ or P_tet_ driving *ddcpf1* expression, presumably these plasmids gave rise to higher levels of ‘leaky’ expression of *ddcpf1*. The fact that even the low copy number CRISPRi plasmids were able to strongly repress *rfp* expression, itself expressed from a high‐copy expression vector, in the absence of inducer suggests that the system is very efficient at repressing gene targets even when ddCpf1 and crRNAs are expressed at very low levels. Plasmid pBbS8c‐*ddcpf1‐rfp* displayed the lowest leaky expression and was therefore selected for testing using reverse transcription‐quantitative PCR (RT‐qPCR). When induced, the *rfp* spacer showed a 90(±4)% repression compared to the no spacer control (Table S3) although a 53% reduction in fluorescence was also seen when inducing the no spacer control, as before (Fig. S4), presumably due to toxicity or metabolic burden of expressing ddCpf1.

The efficiency of the system was further tested for a selection of 11 different chromosomal genes, which have previously been targeted in metabolic engineering efforts to improve the production of flavonoids in *E. coli* either through deletion or CRISPRi (Leonard *et al*., 2008; Kim *et al*., 2016; Carbonell *et al*., 2018; Yang *et al*., 2018). Previously it has been shown that the position of the selected spacer/PAM sequence relative to the transcriptional start site has an inverse relationship with the level of repression observed and that spacers designed on the positive strand are more effective (Qi *et al*., 2013). Therefore, spacer sequences of 23 bp were selected at the first PAM sequence (TTTV) inside the 5′ end of each target CDS on the positive strand (Fig. S5), and introduced into plasmid pBbS8c‐*ddcpf1* using PCR.

Cultures of *E. coli* DH5α with each plasmid were induced in early log‐phase followed by harvesting during late log‐phase. Repression of each gene was measured by RT‐qPCR and compared to cells containing a no spacer control. Seven of the genes were successfully repressed by over 90% (up to 98%) with the remaining four genes being repressed between 22 and 78% (Fig. 2B; Table S4). This shows the system is capable of tight repression of target gene transcription but also that some of the designed spacer sequences were not efficient in repressing their targets and other sequences would need to be tested to achieve tight repression. However, if a range of repression levels was desired these spacers allow the option of partial repression.

## Discussion

The genome editing plasmids presented here show good efficiency and reliability; for gene knockouts and insertions the efficiency was over 75% of colonies screened, and for large DNA insertion (> 8 kb) over 35% of colonies screened. The total protocol – from initial transformation of the target strain to isolation of a markerless mutant strain cured of all plasmids – takes only seven working days. This is similar to previous CRISPR‐Cas editing systems (Jiang *et al*., 2015a; Ao *et al*., 2018) and several days faster than standard recombineering, which typically takes 11 working days. Only single gene knockouts/insertions were tested in this study, but CRISPR‐Cas12a systems have been used for multiplex editing for up to four genes simultaneously (Zetsche *et al*., 2017; Ao *et al*., 2018). We would expect our plasmids would offer the same efficiency of multiplexing with minor modifications to the pTF plasmid editing cassette. This would include extending the CRISPR array to add additional spacer and repeat sequences and an additional dDNA sequence. The cassette would be approximately 150 bp larger for each target, not increasing synthesis cost significantly.

Using standard methodologies, genome integration of large DNA fragments (over 3 kb) is known to be troublesome in *E. coli* (Kuhlman and Cox, 2010). Methods for the insertion of increasingly large DNA fragments are now urgently needed, to endow bacterial chassis with new properties, for example by the stable genome integration of new metabolic pathways. Here, we demonstrate insertion of an 8.4 kb fragment in a single step. Using this same system, we have also integrated a 9.8 kb DNA construct (Robinson *et al*., 2020). Larger cargo DNA could presumably be inserted whilst still maintaining acceptable efficiency; this will be explored in future work to determine the trade‐off between insertion size and efficiency. Furthermore, whilst pTF‐*lacZ* facilitates efficient integration at a well‐characterized locus, substituting the pTF‐*lacZ* homologous recombination arms and the CRISPR array spacer sequence would allow integrations at different loci in a single chassis.

CRISPRi facilitates a more rapid approach to modifying a bacterial chassis and, importantly, to target essential genes by inducible gene knockdown. The design of the plasmid and protocols reported herein allows for plasmid modification to switch the target gene in a single cloning step. By following the presented design rules and associated protocols, we were able to rapidly reduce the expression of 8/12 different genes by over 90%, by designing just a single spacer sequence for each target, similar to repression levels previously achieved in other studies (Qi *et al*., 2013; Zetsche *et al*., 2017; Zhang *et al*., 2017). Previous work demonstrated this positional effect but there was always a degree of variability between the level of repression with some sequences under‐ or overperforming (Qi *et al*., 2013). The variability can be related to off‐target effects or sequences obscured by DNA binding proteins (Yarrington *et al*., 2018). The exact reason for 4 of our spacers to underperform (*sucC, hycI, xapR* and *citE*) is currently unclear. We noted that all of these except *citE* have a PAM motif which has a thymine at the −1 position T(TTTV), which is overlapping PAM motifs and could possibly cause some problems in recognition or binding. During high‐throughput screening of PAM efficiency, it was not identified as a problem to have this sequence (TTTTV) (Kim *et al*., 2017), but we advise to not select these PAM sequences, where possible. In the case of *citE*, we are unsure why it was not an effective spacer but could possibly be due to other regulatory proteins binding in this region. It would be prudent to design more than one spacer sequence for any one target followed by screening for effectiveness before commencing phenotyping experiments. Multiple spacer sequences can also be designed at different points in a coding region resulting in a range of repression levels if more resolution is required (Qi *et al*., 2013; Tao *et al*., 2018). Here we demonstrate targeting single gene sequences but similar CRISPRi systems have been previously used for multiplexed gene repression due to the ease of constructing Cas12a guide arrays (Zetsche *et al*., 2017; Zhang *et al*., 2017) and the designs presented here can easily be modified to target multiple genes simultaneously.

The high levels of repression observed when this CRISPRi system was expressed from a low‐copy plasmid suggest that it might be possible to transfer an optimized CRISPRi cassette from the plasmid vector into the *E. coli* genome for stable long‐term gene regulation, whilst maintaining repression levels. This is an important consideration for engineered strains to remove the need for antibiotics and to provide increased genetic stability, in particular for applications that require the engineered microbes to function over extended periods of time, for example during lengthy industrial fermentations or in medical devices.

CRISPR systems have clearly been demonstrated to be powerful engineering tools and the CRISPR toolset described here has been designed to be easy to use and manipulate, whilst maintaining high efficiency and activity in *E. coli*. Each of the key plasmids described are available from Addgene (www.addgene.org; IDs 153034‐153039), and detailed user protocols are included in the supplementary data (Supporting Information). We have, thus, created and demonstrated a user‐friendly CRISPR toolset, which is easily accessible and affordable to any laboratory.

## Experimental procedures

### Bacterial strains and media

All *E. coli* strains were routinely grown in Lysogeny Broth (LB) or on LB agar plates including antibiotic supplements as appropriate (100 μg ml^‐1^ ampicillin; 100 μg ml^‐1^ hygromycin; 20 μg ml^‐1^ chloramphenicol; 150 μg ml^‐1^ streptomycin). Cloning and plasmid propagation was performed using *E. coli* DH5α (NEB). All bacterial strains used or created in this study are listed in Table S5.

### Construction of plasmids

pSIM*cpf1* was constructed by modification of pSIM18 (Chan *et al*., 2007) to add a codon‐optimized copy of *Acidaminococcus sp*. BV3L6 *cpf1* (Rousset *et al*., 2016; Swainston *et al*., 2018) expressed from constitutive promoter BBa_J23151, and an arabinose‐inducible Cpf1 CRISPR array incorporating two spacer sequences targeting the pMB1 origin of replication. pTF was constructed by modifying pTargetF (Qi *et al*., 2013) to replace the Cas9 sgRNA cassette with a Cas12a CRISPR array using a synthetic DNA fragment. pTF‐*lacZ* was constructed by modifying pTF to add a synthetic DNA containing a double CRISPR array, targeting two PAMs in the *E. coli lacZ* gene, and 500 bp homologous arms flanking these sequences. An *rfp* gene downstream of promoter P_JS23100_ with flanking terminators was cloned between two homologous arms. The fragment was designed to have 15 bp prefix (actctagagaattca) and suffix (atctacaagagtaga) sequences, which allow direct cloning into the pTF vector linearized by inverse PCR with primers pTFopen‐F/R via InFusion cloning (Takara Bio Inc.). CRISPRi plasmids were constructed by cloning synthetic DNA fragments consisting of *ddcpf1* A993A and downstream CRISPR array (targeting *rfp*) driven by a P_trc_ promoter, into plasmids pBbS8c‐RFP, pBbA8c‐RFP, pBbS2c‐RFP, pBbA2c‐RFP, pBbS6c‐RFP and pBbA6c‐RFP^33^. RFP reporter plasmids were constructed by PCR amplification of 198 bp of target gene ORFs using primers with 15 bp overhangs (Table S1) for InFusion cloning into plasmid pBbE11a‐RFP PCR linearized with primers pBbrfpopen‐F/R.

### Design of spacer sequences and arrays

For gene knockouts, Cas12a PAMs (TTTV) were identified approximately central in the target gene, for knock‐ins they were chosen closest to the desired point of integration, on either strand. For CRISPRi, PAMs were selected as the first inside the 5ʹ end of the ORF on the positive strand. Once the PAM was identified, the spacer was designed as the 23 bp immediately downstream on the same (positive) strand.

### Introduction of CRISPR arrays and dDNA to pTF

Arrays were designed and commercially synthesized as part of a cassette which also included any required dDNA (Fig. S1). pTF was PCR linearized with primers pTF‐open‐F/R (Table S1) using CloneAmp polymerase and the cassette cloned using InFusion Cloning (Takara Bio Inc.).

### Introduction of cargo DNA to pTF‐lacZ

Cargo DNA was PCR amplified using CloneAmp polymerase and primers including prefix (CAGGAATTCCATATG) and suffix (TATAGACCATTCGAG) sequences and then cloned into pTF‐*lacZ*, PCR linearized using primers pTF‐lacZ‐cargo‐F/R (Table S1).

### Introduction of CRISPR arrays to pBbS8c‐ddcpf1

Single or double spacer arrays are introduced to pBbS8c‐*ddcpf1*‐Δ, using inverse PCR with primers designed to include overhangs encoding spacers and a 15 bp complementarity overlap for InFusion cloning. Customizable primer pairs for each plasmid (pddcpf1‐crRNAins‐xxx‐F/R) were designed for this PCR (Table S1).

### CRISPR editing

pSIM*cpf1* was introduced into the target strain and was used to make electrocompetent cells. A single colony was inoculated into 5 ml LB supplemented with 150 µg ml^‐1^ hygromycin and grown overnight at 30°C with shaking. The culture was then sub‐cultured, 1:100 into 100 ml of fresh media and grown at 30°C with shaking until OD_600_ ~ 0.2 was reached whereupon it was decanted into 2 × 50 ml centrifuge tubes and incubated in a water bath at 42°C for 15 min immediately followed by incubation in an ice/water bath for 20 min. Tubes were then centrifuged at 3500 *g* for 10 min at 4°C and then the supernatant disposed of. The pellets were each resuspended in 40 ml of chilled, 10% glycerol and centrifuged again followed by resuspension in 1 ml 10% glycerol and transfer to 1.5 ml centrifuge tubes. Each tube was centrifuged at 9000 *g* for 1 min and resuspended again. This was repeated three further times followed by a final resuspension in 250 µl chilled 10% glycerol. Cells were electroporated with 50 ng of pTF plasmids and plated on LB agar containing 150 µg ml^‐1^ hygromycin and 100 µg ml^‐1^ streptomycin then grown for 24 h at 30°C. Miles and Misra dilution series were used to enumerate CFUs for each transformation. Colonies were screened for the correct mutation using colony PCR with primers designed to flank either side of the recombination sites. To remove both plasmids, a single colony was inoculated into 5 ml LB containing 150 µg ml^‐1^ hygromycin and 0.2% L‐arabinose and incubate at 30°C with shaking to an OD_600_ of ~ 1 before sub‐culturing 1:1000 into LB with 0.2% L‐arabinose and incubate at 37°C, with shaking for 16 h. A 10‐fold dilution series was prepared and 100 µl of the 10^‐5^ and 10^‐6^ dilutions plated onto LB agar with no antibiotics and then incubated for 16 h at 37°C. Typically, 20 colonies were replica plated onto three agar plates containing hygromycin, streptomycin or no antibiotics, to confirm loss of both plasmids.

### Measurements of RFP expression for CRISPRi gene regulation

Plasmids were transformed into *E. coli* DH5α/MG1655 and quadruplet colonies were grown overnight in 0.5 ml of media (containing antibiotics as appropriate) in a 2.2 ml 96‐deepwell block at 30°C with shaking at 850 rpm. Cultures were subcultured 1:100 into a black‐walled microtitre plate (Greiner; 655096) containing fresh media (200 µl) with or without supplementation with IPTG (100 μM), L‐arabinose (10 mM) and anhydrotetracycline (200 nM). Plates were sealed with a Moisture Barrier Seal (4titude; 4ti‐0516), then incubated at 30°C with shaking at 850 rpm for 16 h. Readings were taken using a CLARIOstar plate reader (BMG Biotech) for absorbance (600 nm) and RFP fluorescence (584 nm excitation, 607 nm emission).

### RNA extraction

pBbS8c‐*ddcpf1* variants were transformed fresh into *E. coli* DH5α and triplicate colonies were grown overnight in LB (chloramphenicol) and used to inoculate 5 ml fresh media at a 1/50 dilution into 50 ml conical tubes. Cultures were grown at 37°C, with shaking at 180 rpm, to early log‐phase OD_600_ = 0.2–0.4 whereupon they were induced with 10 mM L‐arabinose and 0.1 mM IPTG and growth continued. When the OD_600_ reached 1‐1.4 (1 h) cultures were centrifuged at 21 000 *g* for 1 min, the supernatant discarded and the pellet snap frozen in liquid nitrogen. RNA was extracted using the TRIzol Plus RNA Purification Kit (Thermo Fisher, Waltham, MA, USA) and DNase treated using the PureLink DNase Set (Thermo Fisher). Cell pellets were resuspended in 1 ml of TRIzol and then the method carried out as described by the manufacturer before elution in 50 µl. Each sample was analysed by capillary electrophoresis using a Bioanalyser to determine their RNA integrity numbers (RIN) and 260/280 ratios were determined using a Nanodrop (Thermo) (Table S6).

### Reverse transcription‐quantitative polymerase chain reaction (RT‐qPCR) analysis

First‐strand cDNA was synthesized from 100‐200 ng of total RNA primed with random hexamers using the SuperScript IV First‐Strand Synthesis System (ThermoFisher) according to the manufacturer’s instructions. Oligonucleotide primers for qPCR were designed using the IDT PrimerQuest tool and are listed in Table S7, including amplicon lengths. qPCR was performed using SsoAdvanced Universal SYBR Green Supermix (Bio‐Rad Laboratories Ltd., Watford, UK) in 20 μl reactions in a CFX Connect Real‐Time PCR System (Bio‐Rad Laboratories Ltd) following the manufacturer’s protocol. Quantitative PCR cycle parameters were as follows: initial denaturation at 98°C for 3 min, followed by 40 cycles of 10 s denaturation at 98°C and 30 s annealing and extension at 57°C. Amplification specificity was confirmed by melt curve analysis following qPCR (Fig. S6) and agarose gel electrophoresis. All reactions were performed in technical triplicates. Quantification cycles (*C*_q_) were calculated using software CFX Manager Version 3.0 (Bio‐Rad Laboratories Ltd). Expression of target genes was normalized to reference genes *hcaT* and *idnT* (Zhou *et al*., 2011).

## Funding Information

This work was supported by the UK Biotechnology and Biological Sciences Research Council (BBSRC) and the Engineering and Physical Sciences Research Council (EPSRC) under grants: ‘Centre for synthetic biology of fine and speciality chemicals (SYNBIOCHEM)’ (BB/M017702/1) and ‘Future Biomanufacturing Research Hub’ (EP/S01778X/1). EKRH was supported by the European Union’s Horizon 2020 Research and Innovation Programme under Grant Agreement No. 720793 TOPCAPI—Thoroughly Optimised Production Chassis for Advanced Pharmaceutical Ingredients.

## Author contributions

AJJ designed the plasmids. AJJ, EKRH, MSD and CJR carried out all experimental work. AJJ, EKRH, MSD, CJR, ET and NSS contributed to writing the manuscript. ET and NSS supervised all aspects of the work and secured funding for the study.

## Conflicts of interests

The authors declare no conflicts of interest.

## Supporting information


**Fig. S1**. Sequence of pTF insert cassette.
**Fig. S2**. Testing rfp integration at the ahpC locus using CRISPR‐Cas12a.
**Fig. S3**. PCR screening strategy for genome integration at the lacZ locus.
**Fig. S4**. Example designs of CRISPR‐Cas12a spacer regions for CRISPRi.
**Table S1**. Common PCR primers used in this study.
**Table S2**. Array spacer sequences.
**Table S5**. Plasmids and strains used in this study.
**Table S6**. RNA quality control data.
**Table S7**. Oligonucleotide primers used for qPCR analysis.

**Table S3**. Array spacer sequences.

**Table S4**. Array spacer sequences.
