# Supplementary material for: A plasmid toolset for CRISPR‐mediated genome editing and CRISPRi gene regulation in Escherichia coli
Source: Microb Biotechnol. 2021 Mar 12;14(3):1120–9. doi: 10.1111/1751-7915.13780 (PMC8085919; doi:10.1111/1751-7915.13780)
Supplement: Supplementary file 1 — Fig. S1. Sequence of pTF insert cassette. Fig. S2. Testing rfp integration at the ahpC locus using CRISPR‐Cas12a. Fig. S3. PCR screening strategy for genome integration at the lacZ locus. Fig. S4. Example designs of CRISPR‐Cas12a spacer regions for CRISPRi. Table S1. Common PCR primers used in this study. Table S2. Array spacer sequences. Table S5. Plasmids and strains used in this study. Table S6. RNA quality control data. Table S7. Oligonucleotide primers used for qPCR analysis. [file MBT2-14-1120-s002.pdf]

Supporting Information 1

A plasmid toolset for CRISPR-mediated genome editing and CRISPRi gene regulation in *Escherichia coli*

Adrian J. Jervis[1], Erik K.R. Hanko[1], Mark S. Dunstan[1], Christopher J. Robinson[1], Eriko Takano[1*] and Nigel S. Scrutton[1*].

[†]Manchester Centre for Fine and Speciality Chemicals (SYNBIOCHEM), Manchester Institute of Biotechnology, University of Manchester, Manchester M1 7DN, United Kingdom.

Contents

S1. Sequence of pTF insert cassette 3

S2. Testing *rfp* integration at the *ahpC* locus using CRISPR-Cas12a 4

S3. PCR screening strategy for genome integration at the *lacZ* locus 5

S4. Example designs of CRISPR-Cas12a spacer regions for CRISPRi 6

Table S1. Common PCR primers used in this study 8

Table S2. Array spacer sequences. 9

Table S5. Plasmids and strains used in this study 10

Table S6. RNA quality control data. 12

Table S7. Oligonucleotide primers used for qPCR analysis. 13

S1. Sequence of pTF insert cassette

aggtataatactagt[aatttctactcttgtagat**spacer(23bp)**]$_N$tttttttgaagcttgggcccgaaca
aaaactcggatccaaactcgagtaaggatctccaggcatcaaataaaacgaaaggctcagtcgaaagactgggcctt
tcgttttatctgttgtttgtcggtgaacgctctctactagagtcacactggctcaccttcgggtgggcctttctgcg
tttatacctagggtacg**dDNA**tgaattctctagagt

Figure S1. Sequence of pTF insert DNA cassette. pTF can be modified to target different genes by the introduction of a synthesized cassette with short overhangs for recombination into pTF (underlined) using InFusion cloning. The cassette requires only a CRISPR array (bold, squared brackets) and any desired dDNA (dDNA; bold) sequence for insertion. dDNA typically consists of two homologous recombination arms of 50 bp each for a gene knockout. There are also two transcriptional terminators separating the array and dDNA (italics).

S2. Testing *rfp* integration at the *ahpC* locus using CRISPR-Cas12a

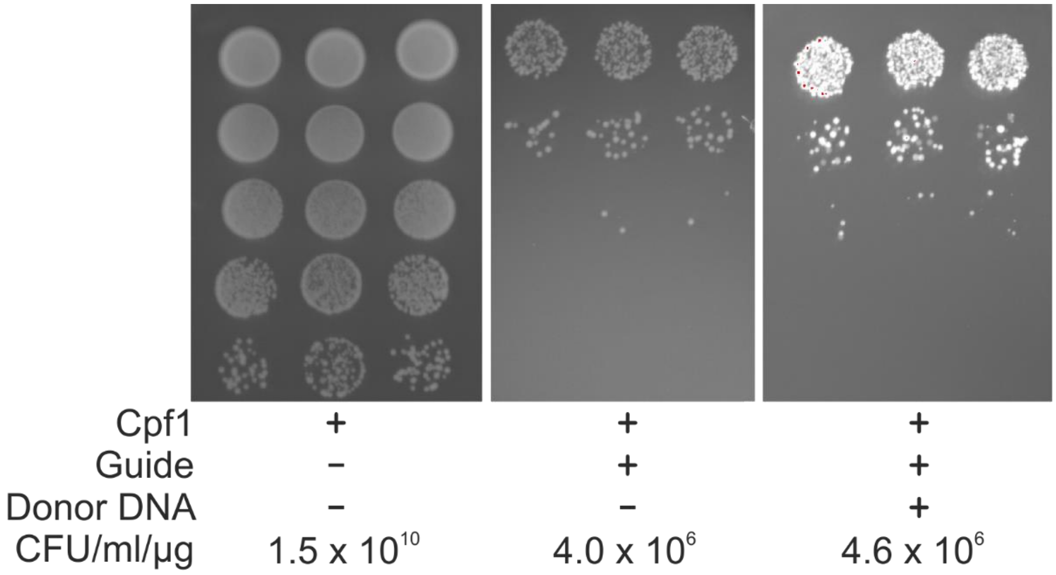

Figure S2. Integration of an in-frame *rfp* reporter into the *ahpC* gene of *E. coli* MG1655. Lambda Red and Cpf1 competent cells were transformed with pTF (no guide, no dDNA), pTF-*ahpC* (*ahpC* guide, no dDNA) or pTF-*ahpC-rfp* (*ahpC* guide and *rfp* dDNA). Dilution series were plated onto agar and after growth, colonies plates were scanned for RFP fluorescence. Comparable CRISPR-mediated cell death could be observed, as CFU counts, with or without dDNA; however, the majority of cells supplied with *rfp* reporter dDNA were fluorescent. PCR analysis confirmed that 86 % of clones supplied with dDNA had undergone correct integration of *rfp* at the *ahpC* locus.

S3. PCR screening strategy for genome integration at the *lacZ* locus

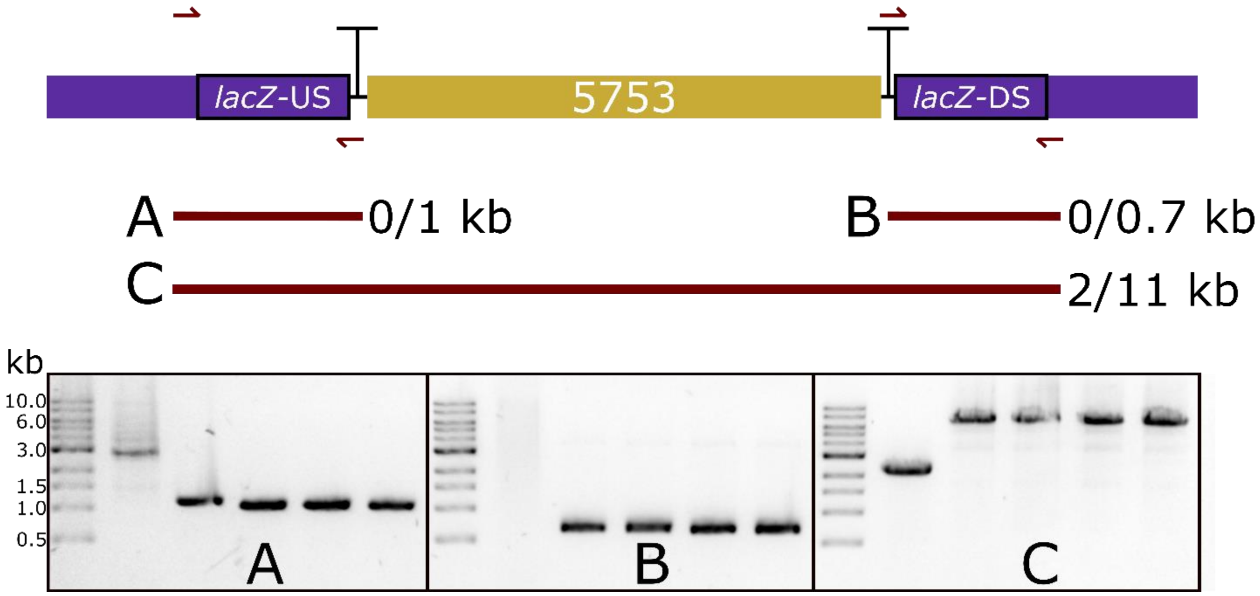

Figure S3. PCR confirmation of genome integration of cargo DNA at the *lacZ* locus. Cargo DNA of construct SBC005753 (yellow) was integrated at the *lacZ* locus (purple) and correct integration was tested using diagnostic PCR with the indicated primers (red arrows) for the 5' (A) and 3' (B) integration sites and the full length integration (C). Primers were designed on the genome flanking the up- and downstream homologous arms (*lacZ*-US and *lacZ*-DS, respectively) and internal to the cargo DNA. Template DNA was purified genomic DNA from a wild-type strain (1st lane in each panel) and 4 potential clones. Predicted sizes of PCR products for wild-type/integrants are displayed above the agarose gel of PCR products. NOTE: some spill-over of DNA ladder can be seen in the first sample lane of panel A.

S4. Repression of *rfp* expression from plasmid pBbE11a-*ddcpf1-rfp* is effective but toxic.

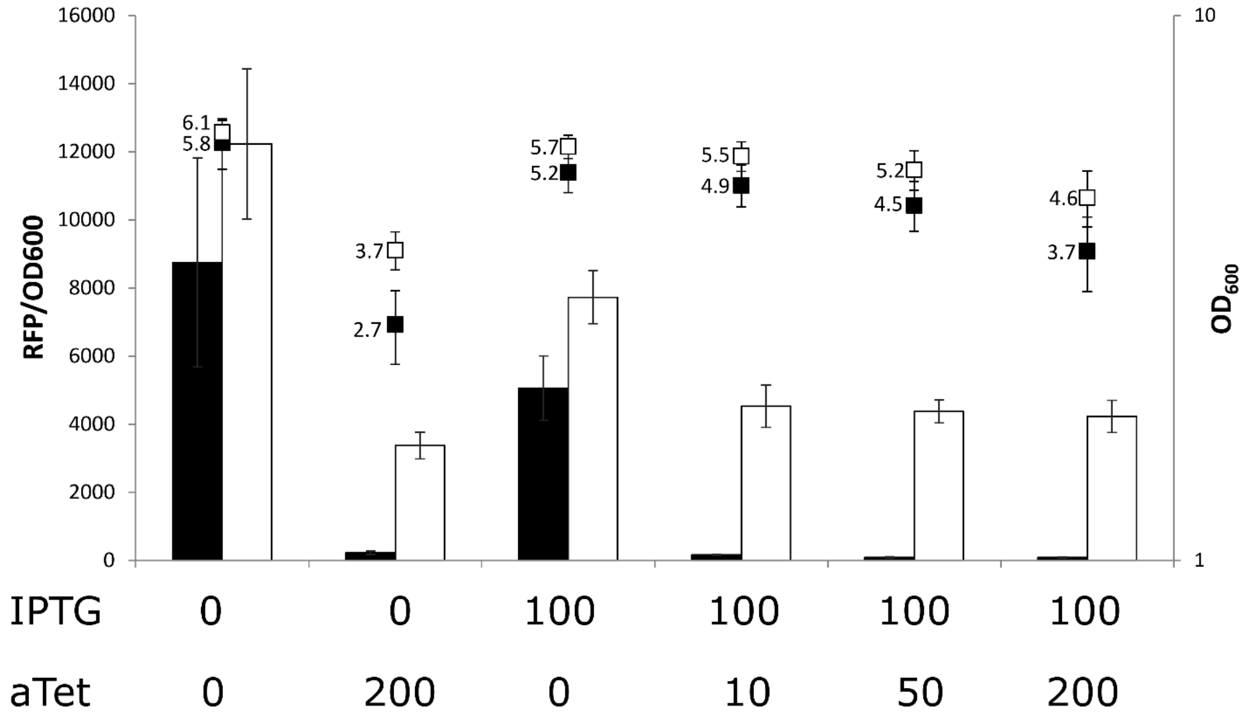

Figure S4. Repression of *rfp* expression from plasmid pBbE11a-*ddcpf1-rfp* is effective but toxic. A ddCpf1 CRISPRi system in plasmid pBbE11a, targeting *rfp* (filled black) or with no spacer sequence (unfilled) were co-expressed with a constitutive *rfp* expression plasmid (25-30 copies) in *E. coli* DH5α. The CRISPR array is under control of the P_{trc} promoter and the *ddcpf1* gene is under control of the P_{tet} promoter. Cultures were induced with different concentrations of the inducers (IPTG and anhydrotetracycline) and RFP fluorescence and culture absorbance (600 nm) were monitored after 24 h at 30 °C. Each sample was grown in biological triplicates and error bars represent the standard deviation.

S4. Example designs of CRISPR-Cas12a spacer regions for CRISPRi

hycI

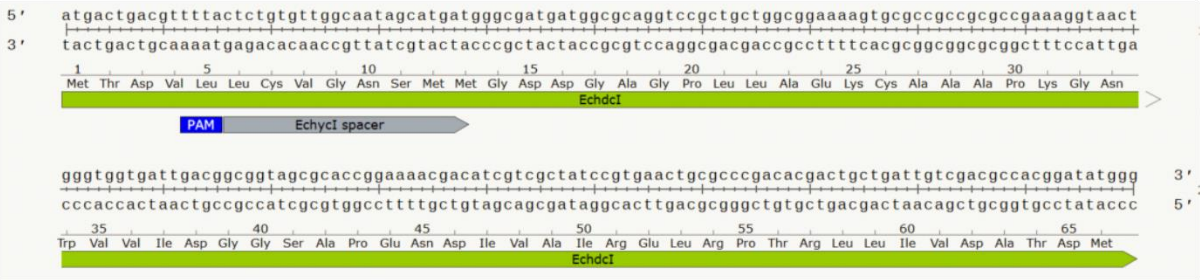

fadR

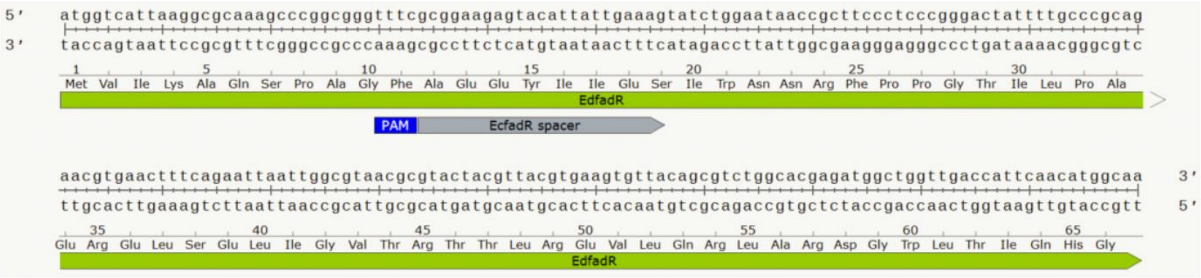

fabF

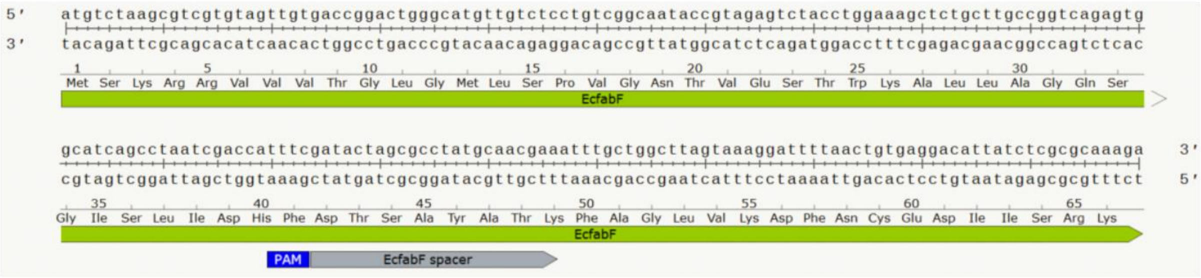

Figure S4. PAM selection and spacer design for CRISPRi applications. The first 200 bp of three example target genes are shown. The first PAM sequence (TTTV) was identified and the23 bp immediately downstream on the positive strand was selected as the spacer sequence to be introduced into CRISPR arrays.

Table S1. Common PCR primers used in this study

Primer	Sequence (5' to 3')	Function
pTFopen-F<br>pTFopen-R	TGAATTCTCTAGAGTCGACCTGC<br>ATCTACAAGAGTAGAAATTACTAGTATTATACCTAGGAC	PCR linearize pTF for cassette insertion
pddcpf1-crRNAins-xxx-F<br>pddcpf1-crRNAins-R	TCTACTCTTGTAGAT$\mathbf{N_{23}}$TTTTTTTGAAGCTTGGGCCCG<br>ATCTACAAGAGTAGAAATTGAAATTGTTATCCGC	PCR linearize pBbS8c-*ddcpf1* for 1 spacer insertion
pTF-lacZ-cargo-F<br>pTF-lacZ-cargo-R	ATATGGAATTCCTGCAGTGC<br>CTCGAATGGTCTATATCCTACG	PCR linearize pTF-*lacZ* for cargo DNA insertion
pBbrfpopen-F<br>pBbrfpopen-R	ATGGCGAGTAGCGAAGACG<br>CATATGTATATCTCCTTCTTAAAAGATCTTTTGAATTCG	PCR linearize pBbE11a-RFP for translational fusions

Table S2. Array spacer sequences.

Gene targets	Array sequence
rfp	*aaagttcgtatggaaggttccgt
pMB1 origin	*tcatagctcacgctgtaggtatc*ccggatcaagagctaccaactct
lacZ	*cgacgttcagacgtagtgtgacg*acgccgtgcgctgttcgcattat
tyrR	*TGAAGACCGACTCGGTCTGACCC
fabF	*gatactagcgcctatgcaacgaa
fabB	*cagcatcggtaataaccagcagg
fabH	*gaaaaaatggtggacacctctga
fadR	*gcggaagagtacattattgaaag
adhE	*actcaagagcaagtagacaaaat
eno	*gtcggtatggcagctgctccgtc
xapR	*cttgccgtagcggaagagttgca
fumC	*cgcatttcgacggagaaaatgcc
sucC	*tgcccgctatggcttaccagcac
hycI	*actctgtgttggcaatagcatga
citE	*cgcttcgctgcaacaacgtaaaa

*Repeat sequence (AATTTCTACTCTTGTAGAT)

Table S5. Plasmids and strains used in this study

**Name**	**Description (Origin of replication, Antibiotic marker, Promoters and Operons)**	**Reference**
pSIM18	Rep101, Hyg^R, P_{PL}-*exo bam beta*	Chan, 2007
pSIM*cpf1**	pSIM18, $P_{JS23151}$ *Ascpf1*, P_{BAD}-α-pMB1 array	This study
pTargetF	pMB1, Spc^R, $P_{JS23119}$-Cas9 sgRNA	Jiang, 2015
pTF	pMB1, Spc^R, $P_{JS23119}$-Cas12a array	This study
pTF-*ahpC*	pMB1, Spc^R, $P_{JS23119}$-*ahpC* spacer	This study
pTF-*ahpC-rfp**	pMB1, Spc^R, $P_{JS23119}$-*ahpC* spacer, *ahpC::rfp* dDNA	This study
pTF-*tyrR-tyrR*	pMB1, Spc^R, $P_{JS23119}$-*tyrR* spacer, Δ*tyrR* dDNA	This study
pTF-lacZ	pMB1, Spc^R, $P_{JS23119}$-*lacZ* array, *lacZ* dDNA	This study
pTF-*lacZ-rfp**	pMB1, Spc^R, $P_{JS23119}$-*lacZ* array, *lacZ::*$P_{JS23100}$-*rfp* dDNA	This study
pTF-*lacZ*-5753	pMB1, Spc^R, $P_{JS23119}$-*lacZ* array, *lacZ::*5753	This study
pBbS8c-RFP	SC101, Chl^R, P_{BAD}-*rfp*	Lee, 2013
pBbA8c-RFP	p15a, Chl^R, P_{BAD}-*rfp*	Lee, 2013
pBbS2c-RFP	SC101, Chl^R, P_{tet}-*rfp*	Lee, 2013
pBbA2c-RFP	p15a, Chl^R, P_{tet}-*rfp*	Lee, 2013
pBbS6c-RFP	SC101, Chl^R, P_{Llac01}-*rfp*	Lee, 2013
pBbA6c-RFP	p15a, Chl^R, P_{Llac01}-*rfp*	Lee, 2013
pBbE11a-*rfp*	ColE1, Amp^R, $P_{JS23150}$-*rfp*	Jervis, 2019
pBbS8c-*ddcpf1-rfp**	SC101, Chl^R, P_{BAD}-*ddcpf1*, $P_{JS23119}$-*rfp* spacer	This study
pBbA8c-*ddcpf1-rfp*	p15a, Chl^R, P_{BAD}-*rfp*, $P_{JS23119}$-*rfp* spacer	This study
pBbS2c-*ddcpf1-rfp*	SC101, Chl^R, P_{tet}-*rfp*, $P_{JS23119}$-*rfp* spacer	This study
pBbA2c-*ddcpf1-rfp*	p15a, Chl^R, P_{tet}-*rfp*, $P_{JS23119}$-*rfp* spacer	This study
pBbS6c-*ddcpf1-rfp*	SC101, Chl^R, P_{Llac01}-*rfp*, $P_{JS23119}$-*rfp* spacer	This study
pBbA6c-*ddcpf1-rfp*	p15a, Chl^R, P_{Llac01}-*rfp*, $P_{JS23119}$-*rfp* spacer	This study
pBbS8c-*ddcpf1-Δ*	SC101, Chl^R, P_{BAD}-*ddcpf1*, $P_{JS23119}$-no spacer	This study
pBbS8c-*ddcpf1-fabF*	SC101, Chl^R, P_{BAD}-*ddcpf1*, $P_{JS23119}$-*fabF* spacer	This study
pBbS8c-*ddcpf1-fabB*	SC101, Chl^R, P_{BAD}-*ddcpf1*, $P_{JS23119}$-*fabB* spacer	This study
pBbS8c-*ddcpf1-fabH*	SC101, Chl^R, P_{BAD}-*ddcpf1*, $P_{JS23119}$-*fabH* spacer	This study

pBbS8c-*ddcpf1-fadR*	SC101, Chl^R, P_{BAD}-*ddcpf1*, $P_{JS23119}$-*fadR* spacer	This study
pBbS8c-*ddcpf1-adhE*	SC101, Chl^R, P_{BAD}-*ddcpf1*, $P_{JS23119}$-*adhE* spacer	This study
pBbS8c-*ddcpf1-eno*	SC101, Chl^R, P_{BAD}-*ddcpf1*, $P_{JS23119}$-*eno* spacer	This study
pBbS8c-*ddcpf1-xapR*	SC101, Chl^R, P_{BAD}-*ddcpf1*, $P_{JS23119}$-*xapR* spacer	This study
pBbS8c-*ddcpf1-fumC*	SC101, Chl^R, P_{BAD}-*ddcpf1*, $P_{JS23119}$-*fumC* spacer	This study
pBbS8c-*ddcpf1-sucC*	SC101, Chl^R, P_{BAD}-*ddcpf1*, $P_{JS23119}$-*sucC* spacer	This study
pBbS8c-*ddcpf1-hycI*	SC101, Chl^R, P_{BAD}-*ddcpf1*, $P_{JS23119}$-*hycI* spacer	This study
pBbS8c-*ddcpf1-citE*	SC101, Chl^R, P_{BAD}-*ddcpf1*, $P_{JS23119}$-*citE* spacer	This study
SBC005753	SC101, Kan^R, P_{trc} - EcDAHPS(fbr) - P_{lacUV5} - EcCMPDH(fbr) - P_{lacUV5} - EcPEPS	Robinson, 2020
**Strain**	**Genotype**	**Source**
DH5α	F– *endA1 glnV44 thi*-1 recA1 relA1 gyrA96 deoR nupG purB20 φ80dl*acZ*ΔM15 Δ(*lacZYA-argF*)U169, *hsdR17*(rK–mK+), λ–	New England Biolabs
MG1655	K-12 F– λ– *ilvG– rfb*-50 *rph-1*	ATCC
SBC010502	MG1655 *lacZ*::P23200-*rfp*	This study
SBC010789	DH5α Δ*tyrR* Δ*pheAL lacZ*::5753	This study
SBC010788	MG1655 Δ*tyrR* Δ*pheAL lacZ*::5753	This study
MG1655 *ahpC::rfp*	K-12 F– λ– *ilvG– rfb*-50 *rph*-1 *ahpc::rfp*	This study

*Plasmids deposited with Addgene (www.addgene.org).

Table S6. RNA quality control data.

Sample*	Concentration (ng/µl)	RIN	260/280	Sample*	Concentration (ng/µl)	RIN	260/280
**Figure 2A. CRISPRi repression of *rfp***				**Figure 2B. CRISPRi repression of chromosomal genes**			
**Δ1-**	16	8.9	1.77	**Δ1**	16	7.8	1.69
**Δ2-**	93	9.4	1.98	**Δ2**	63	8.5	1.97
**Δ3-**	146	9.8	2.08	**Δ3**	157	9.4	2.07
**Δ1+**	178	9.6	2.04	**fabF1**	15	8.4	1.79
**Δ2+**	205	9.6	2.08	**fabF2**	451	10	2.08
**Δ3+**	175	9.5	2.08	**fabF3**	141	9.6	2.04
**R1-**	103	9.9	2.06	**fabB1**	14	9.3	1.75
**R2-**	158	10	2.06	**fabB2**	16	8.2	1.79
**R3**	187	10	2.06	**fabB3**	66	9.6	1.95
**R1+**	400	10	2.08	**fabH1**	44	9	1.92
**R2+**	107	9.9	2.06	**fabH2**	18	9.4	1.8
**R3+**	625	10	2.04	**fabH3**	81	100	2.04
				**fadR1**	117	8.9	1.91
				**fadR2**	128	9.4	2
				**fadR3**	53	10	1.95
				**sucC1**	30	8.6	1.84
				**sucC2**	49	9.8	1.91
				**sucC3**	12	7.6	1.73
				**fumC1**	12	8.1	1.75
				**fumC2**	77	10	1.21
				**fumC3**	21	8.9	0.47
				**adhE1**	26	9.1	1.88
				**adhE2**	187	10	2.06
				**adhE3**	67	10	2
				**eno1**	58	10	1.9
				**eno2**	27	8.9	1.88
				**eno3**	78	9.9	2.01
				**xapR1**	162	9.1	2.06
				**xapR2**	45	9.9	0.82
				**xapR3**	181	9.4	1.85
				**hycI1**	174	9.4	2.04
				**hycI2**	172	9.3	1.91
				**hycI3**	49	9.7	1.21
				**citE1**	222	9.3	2.07
				**citE2**	19	8.2	1.92
				**citE3**	137	9.1	2.08

*Δ= no guide, number = replicate, +/- = induced/no induction

Table S7. Oligonucleotide primers used for qPCR analysis.

Gene	Accession number	Primer name	Sequence (5' to 3')	Amplicon length
eno	b2779	eno_f	CGCTAACTCCATCCTGATCAAA	115
		eno_r	GCCAGAACGGTGAGAGATAAC	
adhE	b1241	adhE_f	GCTACAATGCGAACGACAAC	93
		adhE_r	GGTCGGCAATTTCAGCATAAC	
fumC	b1611	fumC_f	CGGTGGGTACTGGACTAAATAC	84
		fumC_r	CAAACGGTGCACAGGTAATG	
sucC	b0728	sucC_f	CTCCGCACCTGATCCATAAA	99
		sucC_r	GTTTACCTTCCAGACCCAGTT	
fabB	b2323	fabB_f	GGGTGCGCTGTCTACTAAATAC	116
		fabB_r	GCTCTTCAACCACTACCATACC	
fabF	b1095	fabF_f	CGGTATGCTGGTACTTGAAGAG	114
		fabF_r	GGCGGTGACGTCATATGATAA	
fabH	b1091	fabH_f	CATCAGGCTAACCTGCGTATTA	77
		fabH_r	AGCGTCACCACGACATTATC	
fadR	b1187	fadR_f	CTGGGCTTCTACCACAAACT	102
		fadR_r	CCAAATCTCGCCACTCTCAT	
xapR	b2405	xapR_f	ATTGGCTCGGATTGAACAAATAG	111
		xapR_r	CTGAGGAATCGCCGCATAA	
hycI	b2717	hycI_f	GATGACCCAGCCGATTAAAGA	103
		hycI_r	CTACTCTTCTTCCACCGCTAAC	
citE	b0616	citE_f	CATCAAACAGCTGGGCTTTG	103
		citE_r	GGCGTGATCCACTTCTTTCT	
hcaT	b2536	hcaT_f	CCGTTTACAGGCGGTTTACT	96
		hcaT_r	CCGTGGCCCAGATATTGATAC	
idnT	b4265	idnT_f	GTGCGCCTCTTCTTTGAATTT	104
		idnT_r	TCGATGGTGCGTCCATTAC	
rfp	N/A	rfp_f	CGACGCTGAAGTTAAAACCA	107
		rfp_r	TGTAGTCTTCGTTGTGGGAG	
