# Supplementary material for: A plasmid toolset for CRISPR‐mediated genome editing and CRISPRi gene regulation in Escherichia coli
Source: Microb Biotechnol. 2021 Mar 12;14(3):1120–9. doi: 10.1111/1751-7915.13780 (PMC8085919; doi:10.1111/1751-7915.13780)
Supplement: Supplementary file 2 — Table S3. Array spacer sequences. [file MBT2-14-1120-s001.pdf]

PROTOCOL

CRISPR-Cas12a GENOME INTEGRATION – *E. coli*

Integration of large DNA fragments into the genome

CRISPR editing allows the deletion, insertion or mutation of DNA sequences in vivo, either on the genome or on mobile elements such as plasmids. This is challenging when trying to insert large (>4 kb) of DNA onto the genome in a single step. This is a protocol to allow insertion of over 10 kb in a single integration using two plasmids. One plasmid (pSIM*cpf1*) carries a λ red recombination system and the Cas12a nuclease, Cpf1. A second plasmid (pTF-*lacZ*) encodes a CRISPR RNA (crRNA) array, to target Cpf1 to the *E. coli lacZ* locus, and a cargo DNA sequence which includes homologous lacZ recombination sequences flanking and insulated cloning site to add the desired DNA for integration. When both plasmids are present and induced, the λ red recombination system will replace the cut sites with the provided cargo DNA and Cpf1 will cut the genome (killing the cells) of any cells in which recombination does not occur. This theoretically allows implementation and selection of mutations by live/dead screening without the need of a selection marker.

Note: editing is not 100 % efficient and is affected by the size of the cargo DNA.

DESIGN

pTF-*lacZ* construct design

pTF-*lacZ* includes the homologous DNA sequences for integration at the *lacZ* locus already. The desired cargo DNA sequence should be added by either PCR amplification or commercial synthesis to add 15 bp overhangs (prefix-CAGGAATTCCATATG; suffix-TATAGACCATTCGAG) which will allow InFusion cloning into pTF-*lacZ* which has been linearized by PCR with primers, using InFusion cloning:

pTF-lacZ-cargo-F	ATATGGAATTCCTGCAGTGC
pTF-lacZ-cargo-R	CTCGAATGGTCTATATCCTACG

BUILD

Materials

Ice cold, sterile 10 % glycerol

LB media

LB agar plates with:

- No selection (LBA)
- Hygromycin* (150 µg/ml) (LBAh)
- Streptomycin (100 µg/ml) (LBAs)
- Hygromycin and streptomycin (LBAhs)

*Hygromycin is very toxic. Ensure you read the relevant COSHH and Risk Assessments and take appropriate safety measures. It is recommended to purchase hygromycin solution rather than powder.

Introduction of pSIM*cpf1*

Plasmid pSIM*cpf1* should be transformed into target chassis by standard procedures. NOTE: pSIM*cpf1* has a heat-sensitive origin of replication and strains should be incubated/grown at 30 °C at all times including during transformation recovery.

1. Inoculate single colony of pSIM*cpf1* strain into 5 ml LB + 150 µg/ml hygromycin and grow overnight (O/N) at **30°C**.
2. Back dilute O/N 1:100 into 100 ml LB + 150 µg/ml hygromycin in 500 ml flask. Grow at **30°C** with shaking to OD_{600} = 0.2 (approx. 2 hour).
3. Decant culture into 2 pre-chilled 50 ml centrifuge tubes and incubate in a water bath at **42 °C** for 15 min.
4. Transfer tubes directly to ice/water bath and chill for 20 min. **Keep everything well chilled** on ice from this point forward.
5. Centrifuge at 3,500 x g, 4°C, 10 min.
6. Dispose of supernatant and resuspend each pellet in 40 ml ICE-COLD, STERILE 10 % glycerol.
7. Centrifuge 3,500 x g 10 min and repeat once.
8. Gently resuspend and combine pellets in 1 ml 10 % glycerol and transfer to 1.5 ml microcentrifuge tubes.

9. Centrifuge 9,000 x g, 1 min.

10. Remove supernatant.

11. Repeat steps 8-10 a further 3 times (keep cells on ice between steps).

12. Resuspend in 250 ul ICE-COLD, STERILE 10 % glycerol.

For electroporation:

13. Pre-cool 2 mm gap electroporation cuvettes.

14. Set up following (for each pTarget construct) on ice aseptically in sterile ice-cold Eppendorfs:

    a. 50 ul cells + 50 ng pTF (negative control)

    b. 50 ul cells + 50 ng pTF-*lacZ-rfp* (positive control)

    c. 50 ul cells + 50 ng your pTF-*lacZ* variant

15. Incubate tubes on ice for 5 min and transfer each mixture into a separate pre-chilled electroporation cuvette.

16. Electroporate at 2.5 kV, 200 Ω, 25 μF and immediately add 500 μl SOC media to each and transfer to 1.5 ml microcentrifuge tubes.

17. Incubate at 30°C for 2 hours

18. Plate 100 µl onto LBAhs. A recommendation is to make a Miles and Misra dilution series to enumerate colony forming units (CFUs) and observe efficiency of editing.

19. Incubate O/N at 30 °C

Screening colonies

Your negative control (pTF) plate should have many colonies (>10^8 cfu/ml/ug DNA).

An editing pTF-*lacZ* variant should have several (~3) orders of magnitude less CFUs, due to cleavage of the genomic DNA, but can vary.

Screen clones by PCR if possible to confirm the modification. If the cargo DNA is large it can be difficult to successfully screen using colony PCR and genomic DNA should be extracted from several potential clones to serve as the template DNA. Primers to screen the upstream, downstream and across the whole integration site can be used (Figure 1).

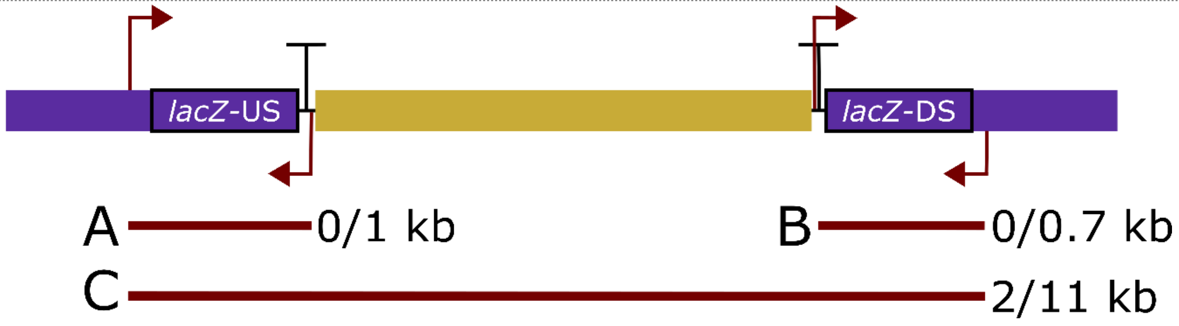

Figure 1. PCR screening strategy for *lacZ* integrants. PCRs A, B, and C are carried out to check the upstream and downstream integration junctions using primers (red) designed in *lacZ* outside the recombination sites in combination with primers designed in pTF-*lacZ*. The size of expected PCR products is shown for wilt-type/mutant. A and B will always be the same size and only produced in mutants (no product in wild-type). The PCR C mutant product will vary depending on the size of the cargo DNA, shown is the predicted size when the cargo DNA is 7.9 kb (not including the homologous arms).

Primers:

lacZflank-F GCTTGCTGCAACTCTCTCAG

lacZflank-R GATGGTTTGCCCGGATAAACG

pTFinternal-F CTCGAATGGTCTATATCCTACG

pTFinternal-R ATATGGAATTCCTGCAGTGC

PCR A: lacZflank-F + pTFinternal-R

PCR B: pTFinternal-F + lacZflank-R

PCR C: lacZflank-F + lacZflank-R

Removal of pSIM*cpf1* and pTF plasmids

Once the desired mutation has been confirmed you must remove the editing plasmids. pSIM*cpf1* has an arabinose-inducible crRNA to cut pTF plasmids and itself has a heat-sensitive origin of replication allowing curing at temperatures of 37 °C or above.

1. Inoculate a single colony to 5 ml LB with hygromycin and 0.2 % L-arabinose and incubate at 30 °C with shaking to OD_{600}~1 (~ 6h).
2. Sub-culture 1/1000 into LB with 0.2 % L-arabinose and incubate at 37 °C, with shaking overnight.
3. Prepare 10-fold dilution series and plate 100 µl of the 10^{-5} and 10^{-6} on LBA and incubate at 37 °C overnight.
4. **OPTIONAL** also plate on LBAh and LBAs overnight at 30 °C to compare the number of CFUs as an indicator of curing.
5. Replica plate 20 colonies on LBA, LBAh and LBAs to confirm loss of both plasmids.

Summary protocol

Day 1: Transform strain with pSIMcpf1b

Day 2: Inoculate colony overnight

Day 3: Prepare competent cells and transform editing plasmids

Day 4: Inoculate potential clones for genomic DNA extraction

Day 5: Genomic DNA extraction and PCR confirmation of integration

Day 6: Plasmid curing

Day 7: Replica plating to confirm plasmid loss

Day 8: Clone selection
