# Supplementary material for: A plasmid toolset for CRISPR‐mediated genome editing and CRISPRi gene regulation in Escherichia coli
Source: Microb Biotechnol. 2021 Mar 12;14(3):1120–9. doi: 10.1111/1751-7915.13780 (PMC8085919; doi:10.1111/1751-7915.13780)

Supporting Information 5

A plasmid toolset for CRISPR-mediated genome editing and CRISPRi gene regulation in *Escherichia coli*

Adrian J. Jervis[1], Erik K.R. Hanko[1], Mark S. Dunstan[1], Christopher J. Robinson[1], Eriko Takano[1*] and Nigel S. Scrutton[1*].

[†]Manchester Centre for Fine and Speciality Chemicals (SYNBIOCHEM), Manchester Institute of Biotechnology, University of Manchester, Manchester M1 7DN, United Kingdom.

Figure S6. RT-qPCR primer melt curves

hcaT

Reaction efficiency

Log starting quantity	Cq
0	18.59
-0.602	20.61
-1.204	22.62
-1.806	24.77
-2.408	26.55
-3.010	28.97
-3.612	31.12
-4.214	32.62
R^2	0.9987
Slope	-3.393
Efficiency	1.971
Percent efficiency (%)	97.1

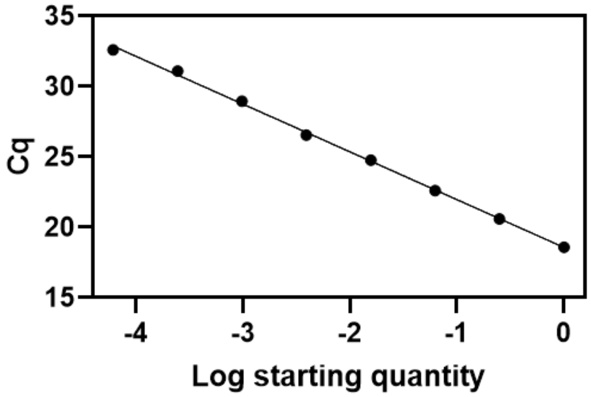

Specificity (melt curve)

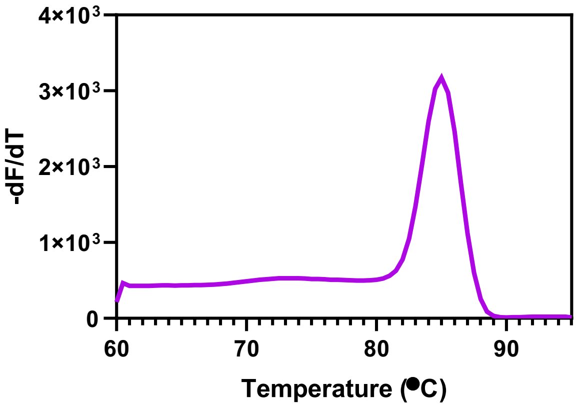

idnT

Reaction efficiency

Log starting quantity	Cq
0	18.70
-0.602	20.73
-1.204	22.77
-1.806	24.86
-2.408	27.08
-3.010	29.13
-3.612	31.21
-4.214	33.04
R^2	0.9997
Slope	-3.442
Efficiency	1.952
Percent efficiency (%)	95.2

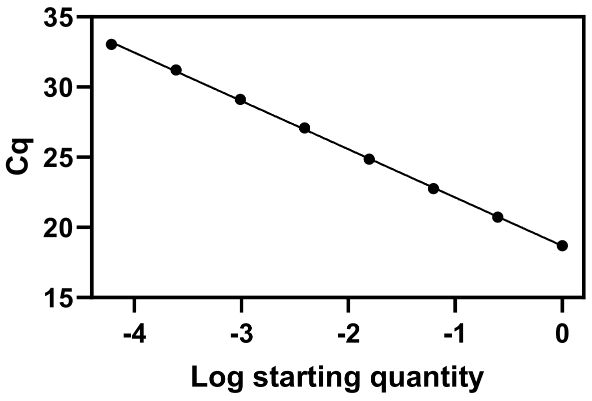

Specificity (melt curve)

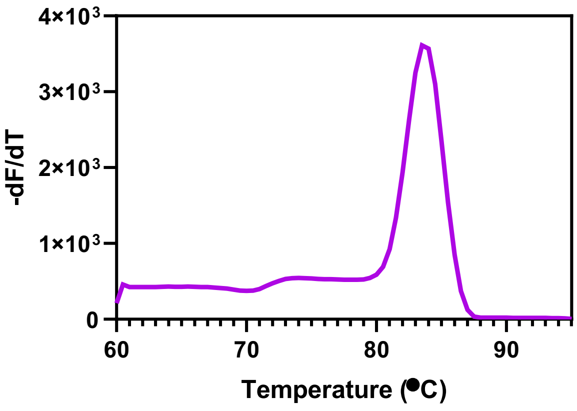

citE

Reaction efficiency

Log starting quantity	Cq
0	17.50
-0.602	19.50
-1.204	21.56
-1.806	23.56
-2.408	25.75
-3.010	27.67
-3.612	29.03
-4.214	32.48
R^2	0.9945
Slope	-3.421
Efficiency	1.960
Percent efficiency (%)	96.0

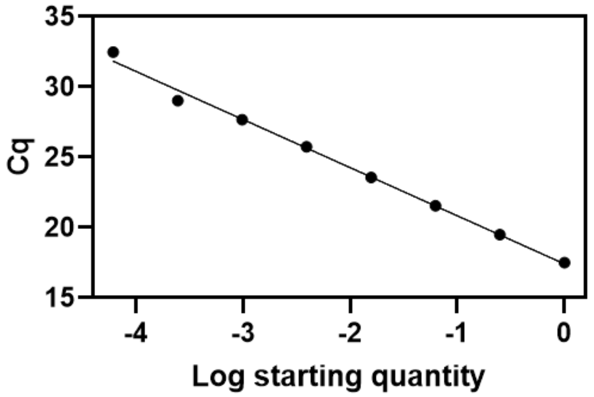

Specificity (melt curve)

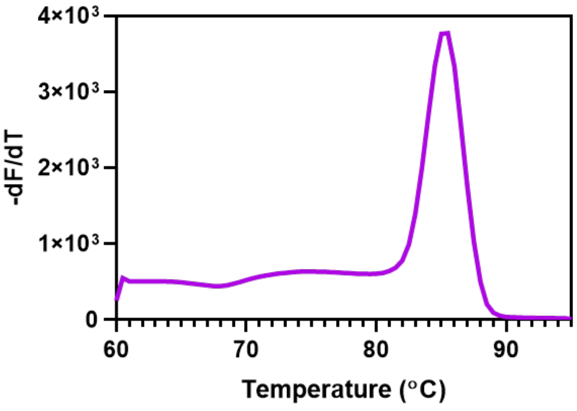

fadR

Reaction efficiency

Log starting quantity	Cq
0	18.62
-0.602	20.64
-1.204	22.64
-1.806	24.63
-2.408	26.80
-3.010	28.90
-3.612	31.15
-4.214	32.56
R^2	0.9987
Slope	-3.383
Efficiency	1.975
Percent efficiency (%)	97.5

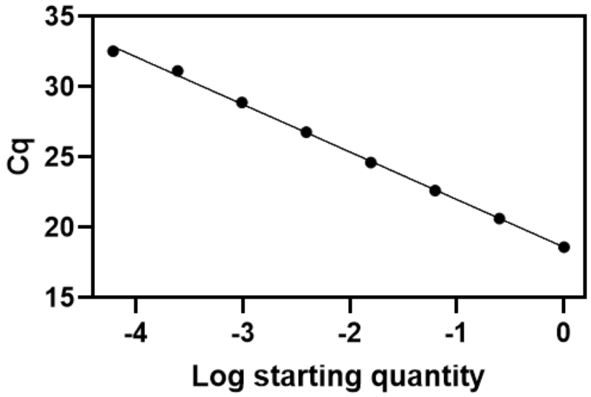

Specificity (melt curve)

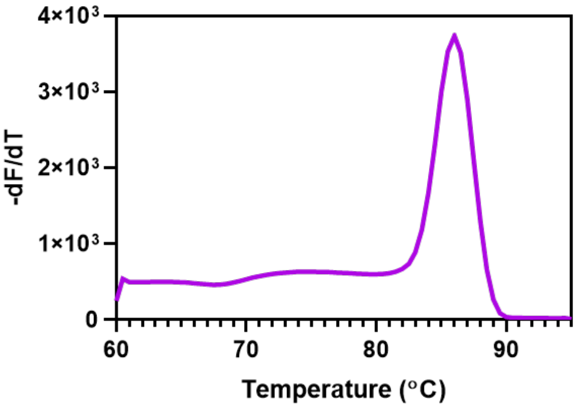

hycI

Reaction efficiency

Log starting quantity	Cq
0	18.36
-0.602	20.49
-1.204	22.63
-1.806	24.72
-2.408	26.87
-3.010	28.81
-3.612	30.99
-4.214	32.71
R^2	0.9994
Slope	-3.433
Efficiency	1.956
Percent efficiency (%)	95.6

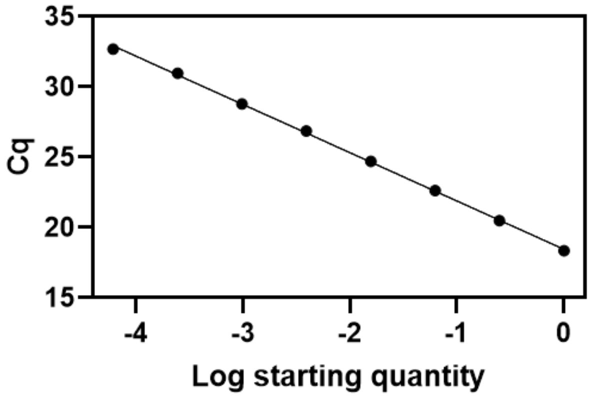

Specificity (melt curve)

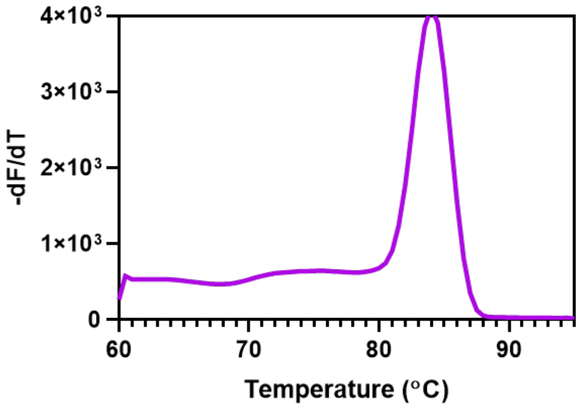

sucC

Reaction efficiency

Log starting quantity	Cq
0	18.62
-0.602	20.66
-1.204	22.89
-1.806	24.94
-2.408	26.97
-3.010	29.24
-3.612	31.67
-4.214	33.04
R^2	0.9984
Slope	-3.501
Efficiency	1.930
Percent efficiency (%)	93.0

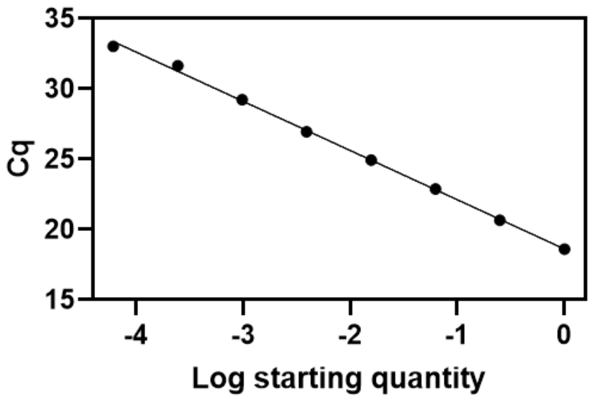

Specificity (melt curve)

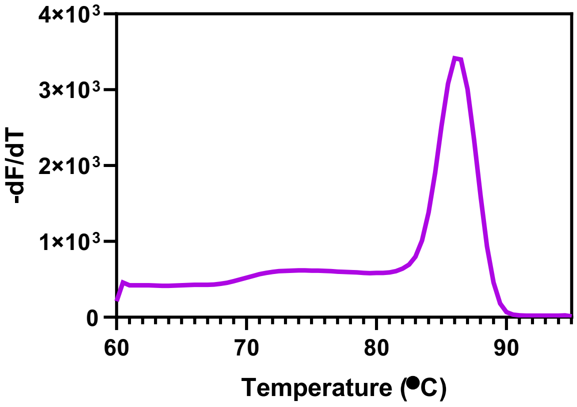

fumC

Reaction efficiency

Log starting quantity	Cq
0	18.64
-0.602	20.70
-1.204	22.94
-1.806	24.84
-2.408	26.84
-3.010	29.06
-3.612	30.92
-4.214	33.06
R^2	0.9997
Slope	-3.407
Efficiency	1.965
Percent efficiency (%)	96.5

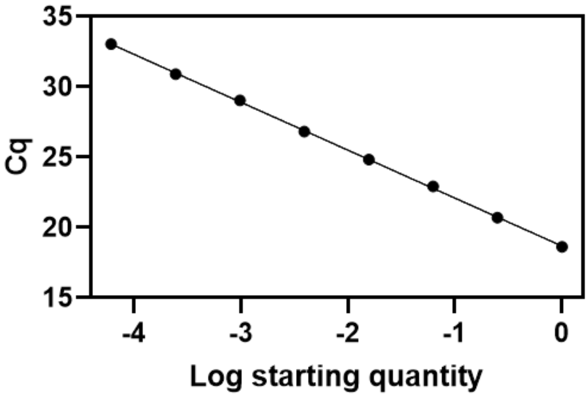

Specificity (melt curve)

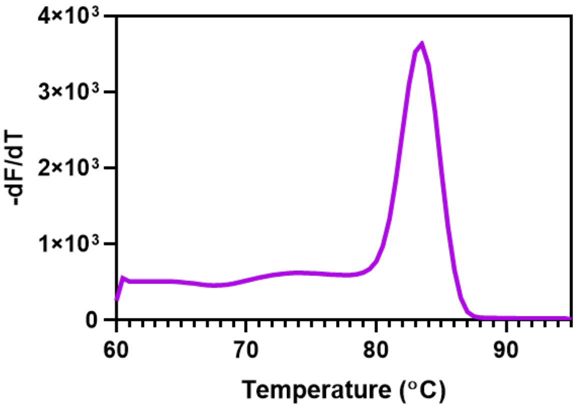

fabB

Reaction efficiency

Log starting quantity	Cq
0	18.27
-0.602	20.40
-1.204	22.60
-1.806	24.75
-2.408	26.87
-3.010	28.87
-3.612	31.01
-4.214	33.15
R^2	0.9999
Slope	-3.522
Efficiency	1.923
Percent efficiency (%)	92.3

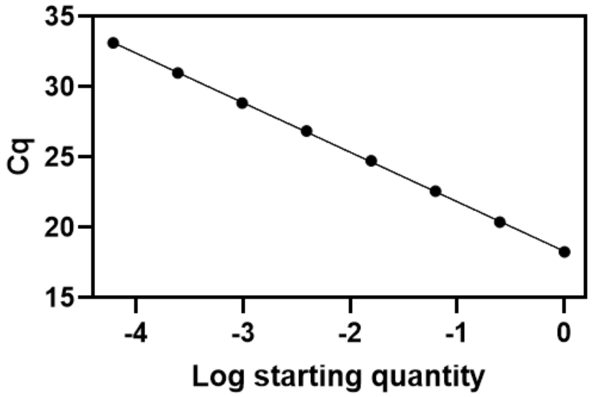

Specificity (melt curve)

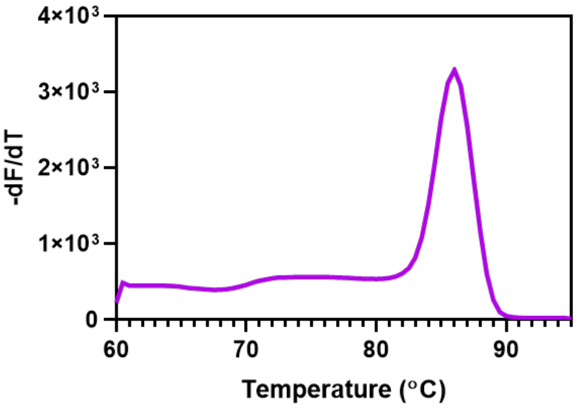

fabH

Reaction efficiency

Log starting quantity	Cq
0	19.21
-0.602	21.30
-1.204	23.47
-1.806	25.53
-2.408	27.66
-3.010	29.65
-3.612	31.72
-4.214	33.21
R^2	0.9985
Slope	-3.377
Efficiency	1.978
Percent efficiency (%)	97.8

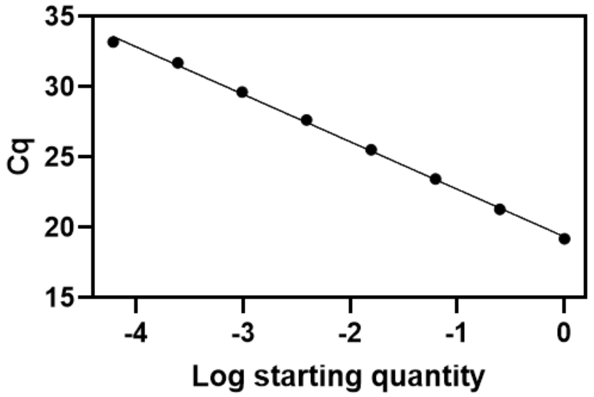

Specificity (melt curve)

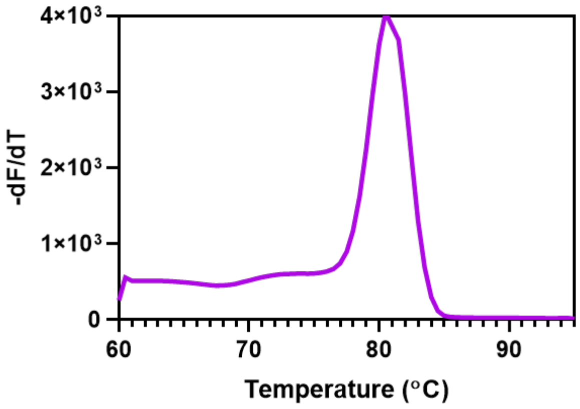

adhE

Reaction efficiency

Log starting quantity	Cq
0	18.48
-0.602	20.51
-1.204	22.74
-1.806	24.84
-2.408	26.76
-3.010	28.85
-3.612	30.69
-4.214	33.13
R^2	0.9994
Slope	-3.435
Efficiency	1.955
Percent efficiency (%)	95.5

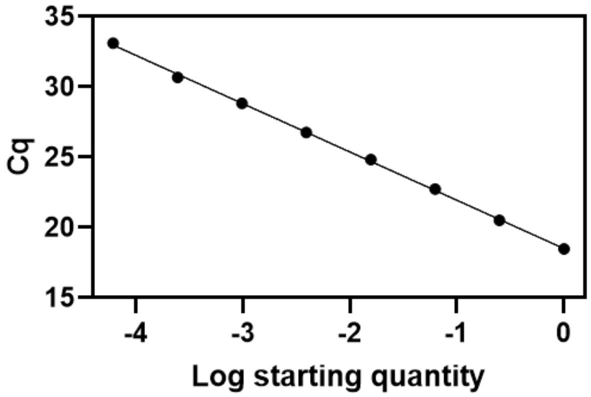

Specificity (melt curve)

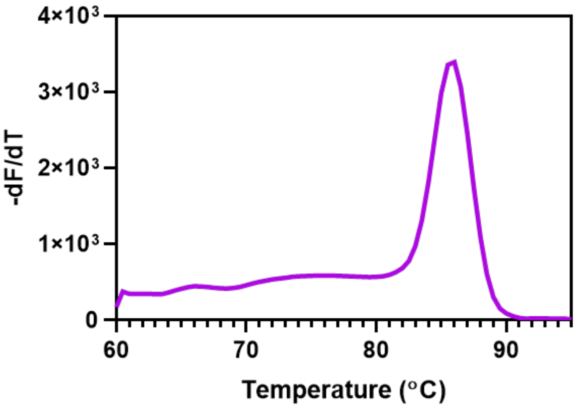

eno

Reaction efficiency

Log starting quantity	Cq
0	18.15
-0.602	20.17
-1.204	22.22
-1.806	24.41
-2.408	26.55
-3.010	28.70
-3.612	29.89
-4.214	32.73
R^2	0.9972
Slope	-3.406
Efficiency	1.966
Percent efficiency (%)	96.6

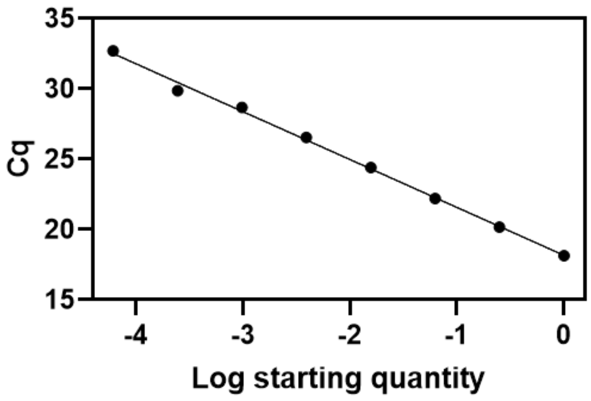

Specificity (melt curve)

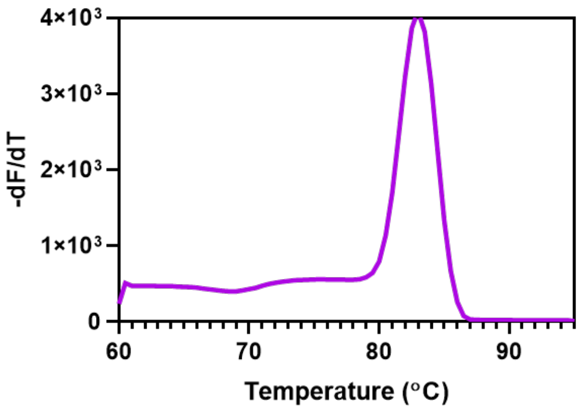

xapR

Reaction efficiency

Log starting quantity	Cq
0	18.38
-0.602	20.42
-1.204	22.58
-1.806	24.70
-2.408	26.79
-3.010	28.76
-3.612	30.12
-4.214	33.13
R^2	0.9969
Slope	-3.409
Efficiency	1.965
Percent efficiency (%)	96.5

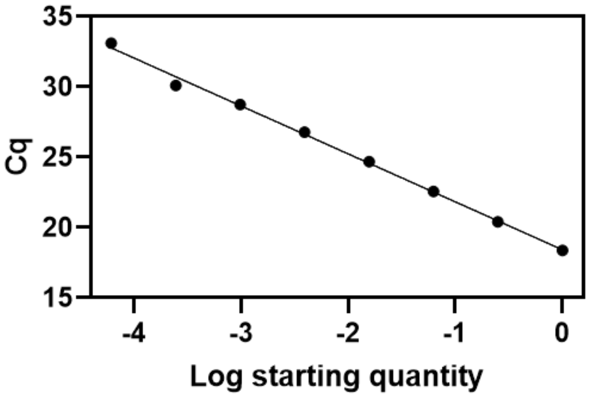

Specificity (melt curve)

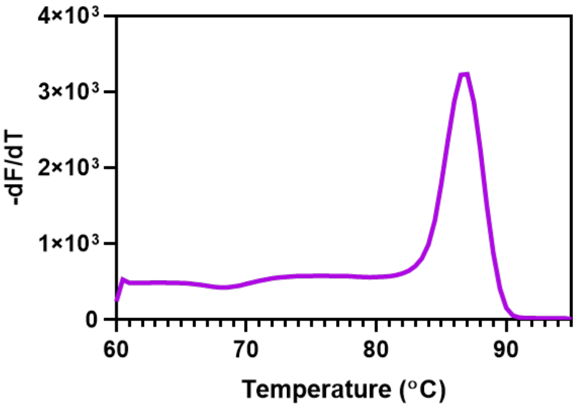

fabF

Reaction efficiency

Log starting quantity	Cq
0	18.49
-0.602	20.54
-1.204	22.70
-1.806	24.73
-2.408	26.84
-3.010	29.05
-3.612	30.65
-4.214	32.54
R^2	0.9988
Slope	-3.363
Efficiency	1.983
Percent efficiency (%)	98.3

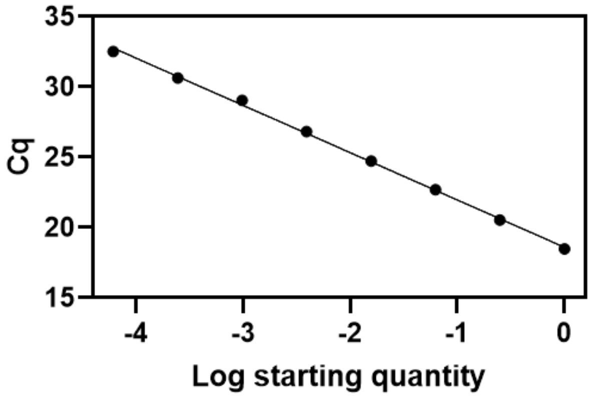

Specificity (melt curve)

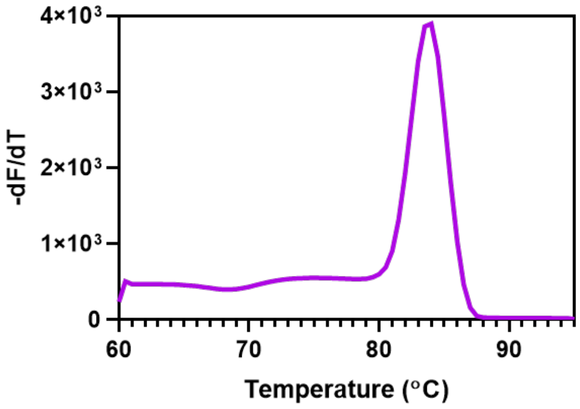

rfp

Reaction efficiency

Log starting quantity	Cq
0	15.16
-0.903	18.19
-1.806	21.34
-2.709	24.42
-3.612	27.64
-4.515	31.02
-5.419	34.15
R^2	0.9998
Slope	-3.517
Efficiency	1.925
Percent efficiency (%)	92.5

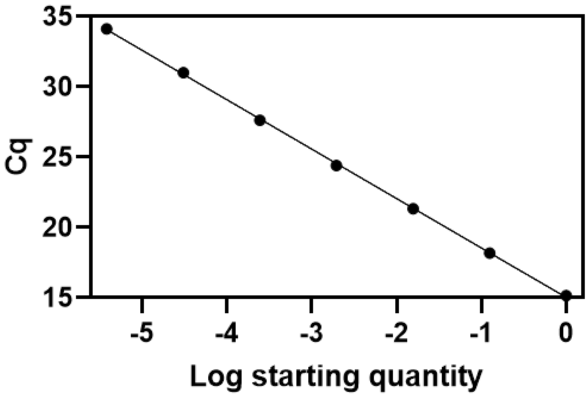

Specificity (melt curve)

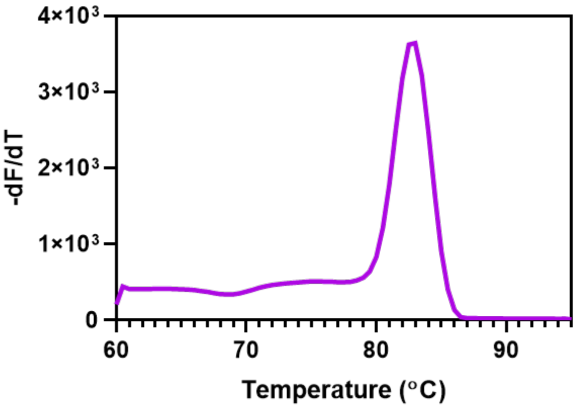

Supplement: Supplementary file 3 — Table S4. Array spacer sequences. [file MBT2-14-1120-s003.pdf]
